# First steps towards a holistic impact assessment methodology for connected and automated vehicles

**DOI:** 10.12688/openreseurope.13870.2

**Published:** 2022-04-11

**Authors:** Diane Cleij, Wendy Weijermars, Rune Elvik

**Affiliations:** 1 SWOV Institute for Road Safety Research, Bezuidenhoutseweg 62, The Hague, 2594 AW, The Netherlands; 2 Institute of Transport Economics, Gaustadalleen 21, Oslo, 0349, Norway

**Keywords:** impact assessment, automated vehicles, connected vehicles, behavioural adaptation, traffic system, societal impacts

## Abstract

Connected and automated vehicles have become more common in recent years, increasing the need to assess their societal level impacts. In this paper a methodology is presented to explore and define these impacts as a starting point for quantitative impact assessment. The many interrelations between impacts increases the complexity of obtaining a complete overview. Therefore, a structured approach is used, which shows many similarities with the modelling of causal-loop-diagrams. Feedback loops between impacts are taken into account at an early stage and both literature review and expert interviews are used to produce a holistic overview of impacts. The methodology was developed and applied in the European H2020 project LEVITATE. The impact taxonomy and interrelations between impacts resulting from this project are presented and further steps needed to perform a quantitative evaluation of the impacts are discussed.

## Introduction

Vehicle automation and connectivity has become more and more common in recent years. Most vehicles on the roads today can take over part of the driving task, such as keeping a constant speed using cruise control or avoiding lane departures using a lane keeping system. Cars with SAE level 2 automation functions, where the driver is only required to monitor the automation, are already being sold and it is expected that conditional, high and full automation functions will become available in the (near) future. While such systems are generally expected to have the potential to increase safety and decrease congestion ( Kockelman *et al.*, 2016), the actual impacts of this technology on a societal level depend on many factors ( Kockelman *et al.*, 2016; Milakis *et al.*, 2017; Sousa *et al.*, 2018).

The European horizon 2020 project LEVITATE aims to offer policy makers insight into the wide range of impacts that vehicle automation can have on society. The policy support tool that will be developed during this project is intended to enable a wide range of policy makers to select policy interventions and assess the impacts of automated vehicles in the short, mid and long term future under different circumstances. To serve this purpose, the first step is to gain an overview of as many of the potential impacts of connected and automated vehicles (CAVs) as possible. A study was therefore made of previous attempts to identify and classify potential impacts of CAVs and the intricate web of interrelations between impacts ( Elvik *et al.*, 2019).

The time scale over which LEVITATE will assess the impacts implies that not only direct, short term, impacts should be considered, but indirect impacts and feedback loops that apply over longer periods should also be included. To obtain such a comprehensive overview of all impacts, a structured holistic approach is needed. E.g., structuring the impact assessment process with the goal of obtaining a holistic set of potential impacts. While many overviews of potential impacts of automated vehicles can be found in the literature ( Chan, 2017; Fagnant & Kockelman, 2015; Hörl *et al.*, 2016; Kockelman & Boyles, 2018), structured holistic approaches to impact assessment of automated vehicles are scarce.

This paper presents the approach taken in LEVITATE to explore and define impacts and their interrelations as a starting point for quantitative impact assessment. The modelling approach shows many similarities with the modelling of causal loop diagrams ( Bala *et al.*, 2017). In the following sections a brief, non-exhaustive list of existing literature on impact analysis of automated vehicles is discussed after which the approach developed within LEVITATE to explore the impacts is presented. The model developed is then presented, containing both direct and indirect impacts and their interrelations that can be easily adapted and extended for specific uses. Finally, the approach is evaluated for different uses and improvements are discussed.

## Review of impact assessment models

Previous projects have proposed overviews of potential impacts of CAVs. Most of these overviews consist of written summaries of impacts discussed in the literature, sometimes enriched with discussions on possible interrelations between these impacts.

In ( Fagnant & Kockelman, 2015) impacts are first discussed under four headings: safety, congestion and traffic operations, travel behaviour impacts and freight transport. Subsequently, they present estimates of societal and personal economic benefits based on literature findings of expected changes in vehicle miles travelled, vehicle ownership, technology cost, crash rates, congestion reduction and parking. In ( Hörl *et al.*, 2016) the impacts of vehicle automation are categorized as impacts on mobility, city planning, car industry, work organisation, user profiles, delivery of goods and price. Within each category many more specific impacts and some interrelations are mentioned.

In ( Chan, 2017) benefits, i.e., positive impacts, of automated vehicles are categorized under vehicle user, transportation operation and society perspectives. Many more overviews can be found in the literature ( Herrmann *et al.*, 2018; Kockelman & Boyles, 2018; Polis, 2018) and are a useful starting point for impact assessment. These overviews, however, do not provide the structured holistic approach to exploring impacts needed to eventually perform a quantitative impact analysis as is the objective of the LEVITATE project.

A more structured approach was taken in ( Milakis *et al.*, 2017), where a comprehensive literature review is presented on the impacts of automated vehicles. They summarize the impacts in a clear and readable model, consisting of four concentric circles showing vehicle automation technology in the centre. The first order impacts of this vehicle technology on the transport system that are directly noticed by the road users are shown around this centre, followed by the second order impacts on, for example, infrastructure and land use in the third circle band. Finally, in the fourth circle band, the wider societal impacts are shown. The model attempts to show the propagation of vehicle technology impacts from direct impacts on road users to societal impacts, giving a more coherent view of the relationship between impacts. While the simplicity of such a model has many advantages, it does not clearly show causal relations between specific impacts that are needed to eventually quantify them.

A very structured approach is taken in ( Innamaa *et al.*, 2018a; Innamaa *et al.*, 2018b). They define nine impact groups that are displayed on a graph of spatial resolution vs. time frame. The direct impacts, those that have a relatively clear cause-effect relationship with the primary activity or action, are those of small spatial resolution and short time frame. These impacts can usually be measured in a field test and are grouped under safety, vehicle operations, personal mobility and energy/emissions. Indirect impacts, on the other hand, are defined as resulting from these direct impacts and can often not be measured in a field test. They include impacts on network efficiency, travel behaviour, public health, infrastructure and land use and socio-economic impacts.

In a first step of their impact analysis approach they perform a classification of the system and the design domain. In this step they, for example, make clear which automated functions and services will be included in the impact analysis. For the impact evaluation they then propose charts indicating potential impact paths starting from direct impacts on vehicle operations, driver or traveller, quality of travel and transport system and leading to one of the previously mentioned impact areas, such as safety. In addition, they recommend not only investigating these one-way paths to the impact areas, but also the strong links between the impact areas. As a next step, they recommend elaborating further on the proposed impact paths for the system under evaluation by adding direction of change, similar to what is done in causal-loop-diagrams.

## Impact assessment method

**Figure 1.**
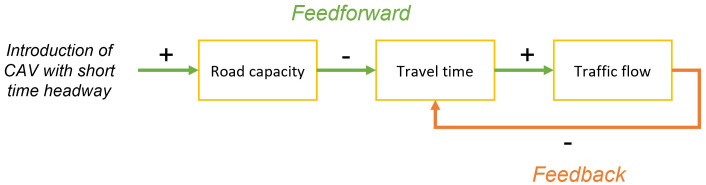
Example of causal relations between impacts, containing both feedforward (green) and feedback relations (orange).

In the LEVITATE project a somewhat different approach has been taken. The focus is put on the system as a whole from the start, thus including both feedforward and feedback casual relations between different impact areas. Examples of such causal relations are shown in Figure 1. Here the feedforward, or direct, relation is the potential impact of CAV regarding the reduction travel time due to the adoption of shorter time headways. The feedback relation is then the relation between increased traffic flow due to this shorter travel time that in turn increases the travel time.

The impact assessment method can be divided into four steps

1. Definition of scope
2. Impact diagram set up
3. Impact diagram elaboration
4. Impact diagram validation

### Initial scoping

The initial definition of scope defined use cases in terms of type of technology (automation, connectivity, mobility as a service) and area of application (passenger cars, urban transport, freight transport). The LEVITATE project focuses on societal level impacts of CAVs in three areas of use: freight, urban and passenger car transport. In Table 1 the LEVITATE scope in terms of more detailed subsystems and technologies within these three areas are shown.

**Table 1.** Example connected and automated vehicles deployment scenarios for each use case.

Use case	Automated urban transport	Passenger cars	Freight transport
**Automation** ** scenarios**	• Point to point shuttle • Anywhere to anywhere shuttle • Segregated pathway operations • On road operations • Intermodal route planning • Street design implications	• SAE L2/3/4 automation • Highway pilot • Autopark • Highway pilot • Cooperative automatic cruise control • Traffic jam pilot • City chauffeur	• Highway platooning • Automated urban delivery • Depot to depot automated transfer • Automated intermodal transport • Synchronized traffic load on bridges • Intelligent access control of infrastructure/bridge
**Connectivity** ** scenarios**	• Green light optimized speed advisory • System-aware route optimization	• Geo-fencing based powertrain use • Green light optimized speed advisory • Road use pricing • System-aware route optimization	• Geo-fencing based powertrain use • Green light optimized speed advisory • Road use pricing • System-aware route optimization
**Mobility as a** ** service**	• Multi-modal integrated payments • e-hailing • Automated ride sharing	• Multi-modal integrated payments • Shared ownership models • Urban platooning	• Local freight consolidation

As the output of the LEVITATE project will be a policy support tool that can be used by municipalities, regional authorities and national governments, impacts on, for example, a European level are outside the impact assessment scope. Finally, the time periods used for the impact assessment are short (five years), medium (10 years) and long term (25+ years). These time periods correspond to the immediate introduction of mobility technologies, the duration of a mixed fleet of non-automated, partial and fully automated vehicles as well as the increase in mobility services based on increasingly ubiquitous connectivity. Within the policy support tool, impacts are estimated for different penetration rates of first and second generation automated vehicles as well as a number of additional policy measures and technologies. The tool will quantify the impacts presented in this paper accordingly.

For example, there are many vehicle-based automation technologies that are close to market. It can be assumed that these will soon enter the vehicle fleet and result in changes compared to current driving. Over the medium term there will be a mixed fleet of vehicles and a range of levels of infrastructure connectivity which may introduce new transport risks, making safety benefits uncertain. Beyond 25 years there will be largely ubiquitous automation with high levels of system integration. Cities are expected to transform as land use, employment and disruptive technologies are expected to cause unexpected changes.

### Impact diagram set up

Setting up the impact diagram started with an explorative literature review on the impacts of CAVs within the scope as defined in the previous paragraph. The review was done using the snowball method through Google Scholar, starting from the paper of Milakis *et al.* (2017) (last search on December 20, 2018). For each study, a list was made of the potential impacts they identified. These lists were then compared. A consolidated list was made from all potential impacts that were mentioned in at least one of the studies that were reviewed. An overview of the impacts described in the found literature (see “ExplorativeLiteratureOverview.pdf” ( Cleij *et al.*, 2021)) was sent to other members of the project and their input was requested. The input from project members was used to update the list of potential impacts from literature.

To visualise these impacts and their interrelations, the impact areas were placed in text balloons and the interrelations between these areas visualized using arrows. The arrowhead indicates the direction of the impact relation, i.e., that changes in travel time will likely impact the commuting distance is indicated with an arrow from the former towards the latter.

To structure the diagram and define a holistic set of starting points generating these impacts, the top of the diagram contains the technological changes that drive the impacts; the impact generators. In the LEVITATE project the following impact generators were defined after some iterations: vehicle design, level of automation and connectivity. All impacts could be derived from these impact generators.

**Figure 2.**
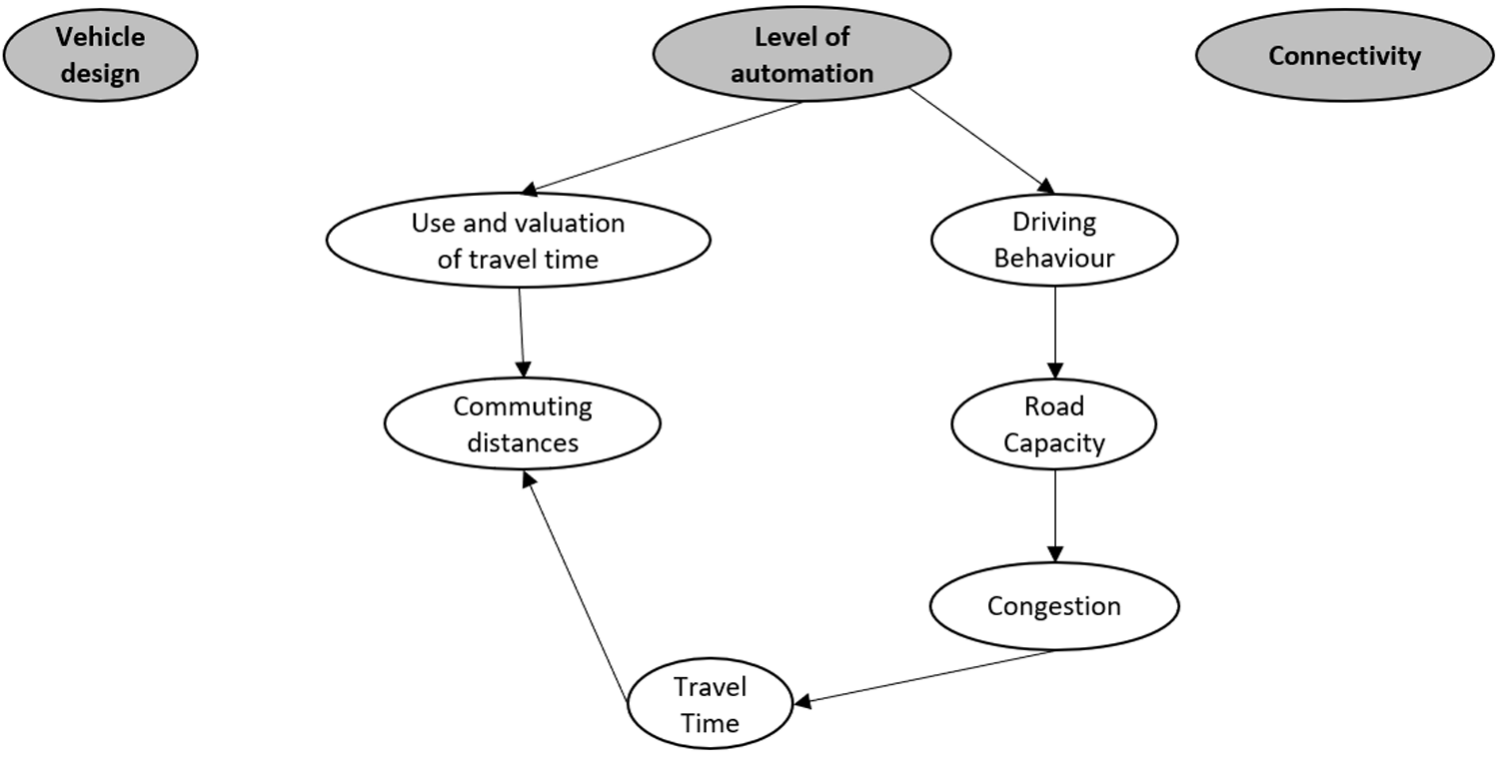
Example of impact diagram set up with three technology areas as impact generators and six possible impact areas and their interrelations.

An example of such an impact diagram set up including six impact areas is shown in Figure 2. This example shows the influence of automation level on the use and valuation of travel time and the driving behaviour (e.g., shorter headways). These in turn influence the commuting distances and road capacity, respectively. The road capacity in turn influenced the congestion, which influences travel time. Travel time in turn, influences commuting distances.

### Impact diagram elaboration

To extend and improve the initial impact diagram, two methods were adopted. Firstly, each impact in the diagram was analyzed for possible further relations to other impact areas in the diagram and impact areas not yet in the diagram. In doing so, additional literature was often consulted. An overview of the most relevant literature used for the development of the impact diagrams can also be found in the underlying data document “OverviewOfMostRelevantLiterature.pdf” ( Cleij *et al.*, 2021).

Second, the impact areas were grouped along dimensions commonly found in the literature. The choice for such dimensions was based on a comparison of impact taxonomies from literature (see Table 2).

**Table 2.** Connected and automated vehicle impact taxonomies from literature.

( Chan, 2017)		( Milakis *et al.*, 2017)	( Polis, 2018)	( Innamaa *et al.*, 2018b)	( Hibberd *et al.*, 2017)
*Main groups*	*subgroups*				
**Vehicle users**	Comfort Convenience Mobility	Travel costs Vehicle ownership and sharing Travel choices Location choices	Travel behaviour	Travel behaviour Personal mobility	Mobility
**Transport** ** operations**		Road capacity Land use Transport infrastructure	Spatial aspects Infrastructure Traffic efficiency	Land use Network efficiency Infrastructure Vehicle operations	Efficiency
**Society**	Environment Energy Economy Safety	Energy consumption Safety Social equity Economy Public health	Road safety Socio-economic	Socio-economic Safety Energy/emissions Public health	Socio-economic Safety Environment

**Figure 3.**
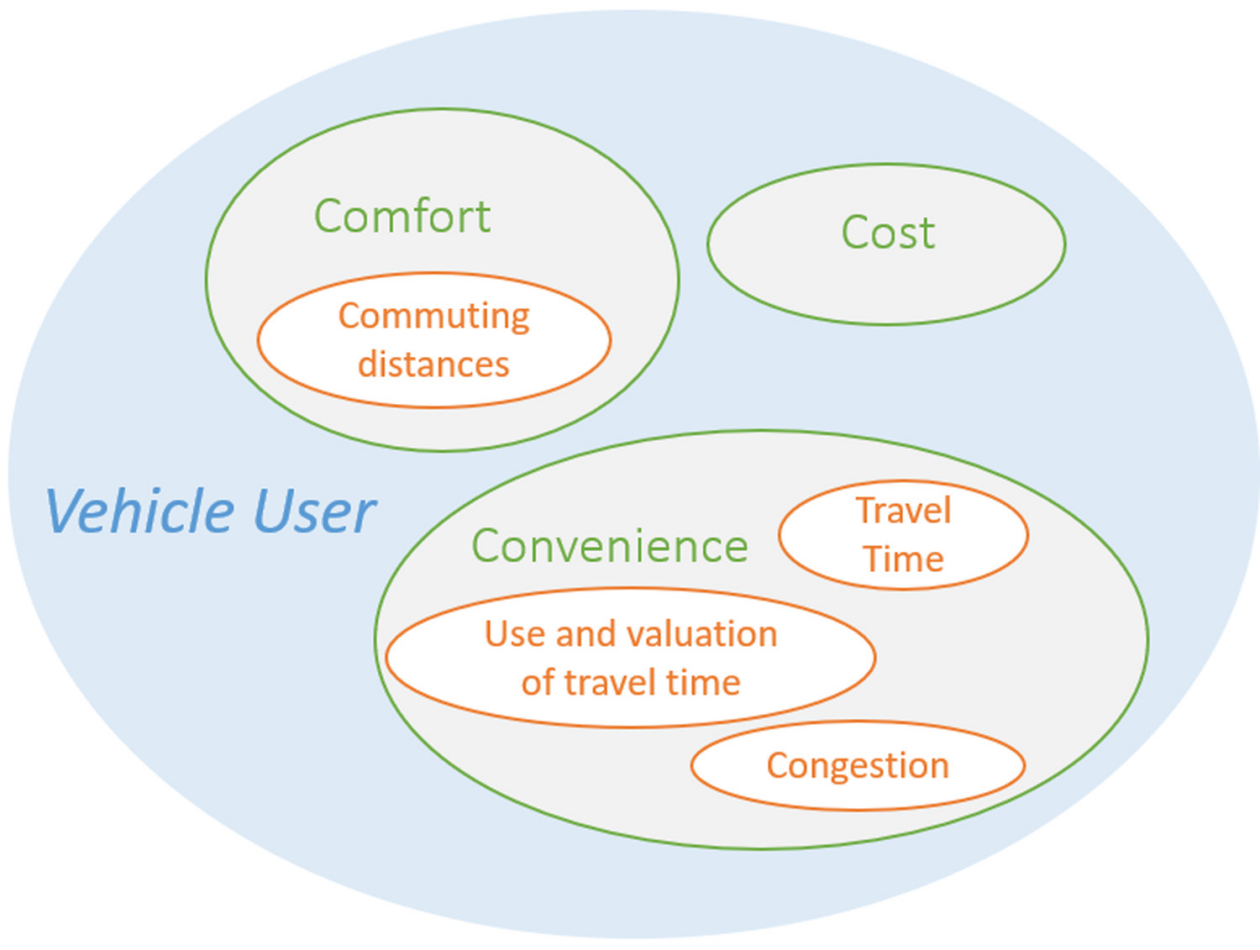
Example of impact area grouping.

The main groups in the taxonomy described in ( Chan, 2017) was deemed most holistic as it encompassed all others. The impacts in this project were therefore classified accordingly, i.e., affecting vehicle users (direct), transportation operations (systemic) and society (wider). In Figure 3 an example is given of such grouping for the impacts from Figure 2 that can be placed in the vehicle user group. To extend the impact diagram, each of these subgroups was analyzed for missing impacts and newly found impacts were added to the overall impact diagram.

Both steps focus on the analysis of impacts from different points of view, making the impact diagram more holistic.

### Impact diagram validation

After several iterations of the impact diagram elaboration step, a final impact diagram was obtained. Whether the diagram includes all potential impacts of CAVs cannot be ascertained at this time. However, the completeness of the diagram is an important objective of the LEVITATE project. Therefore, a validation of the completeness of the diagram was approximated by comparing the impact diagrams to impacts found in additional literature, in combination with a final review by project members. The literature used for this validation ( Litman, 2019; Sousa *et al.*, 2018; van Nes & Duivenvoorden, 2017) was not part of the initial explorative literature review. No additional impacts or interrelations were found and therefore the completeness of the diagram was deemed sufficiently validated.

### Ethics statement

The consultations within this work were performed by other members of the LEVITATE project. Following the grant agreement, these project members consented to use their views.

## Method output: impact model

**Figure 4.**
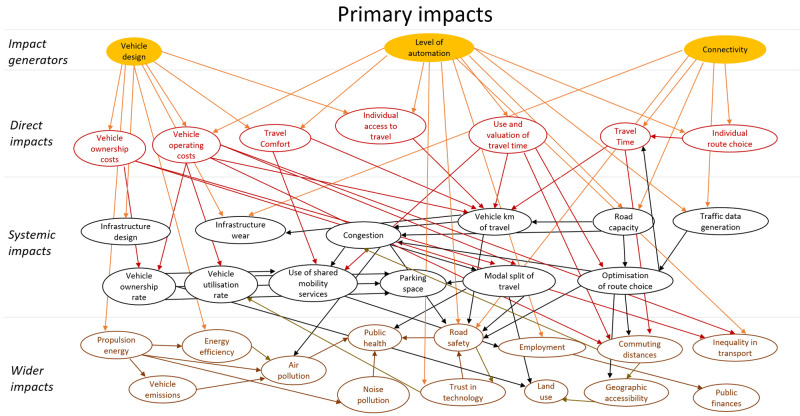
Impact diagram with primary impacts from ( Elvik *et al.*, 2019).

**Figure 5.**
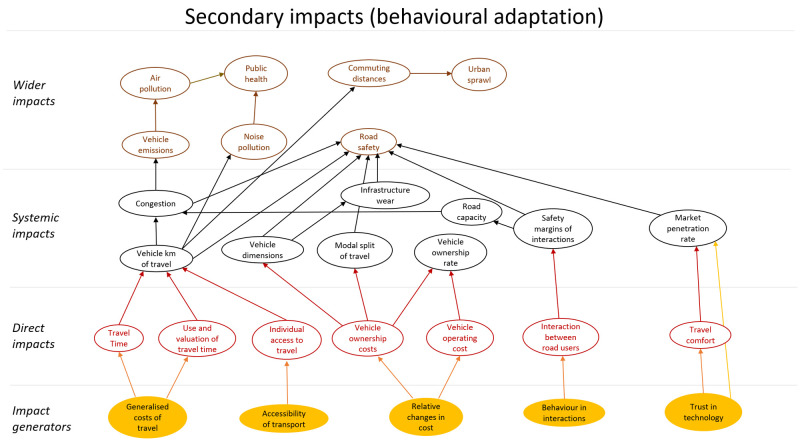
Impact diagram with secondary impacts from ( Elvik *et al.*, 2019).

The final model of impacts is a large complex diagram. To add structure to the diagram a similar approach to the model presented in ( Milakis *et al.*, 2017) was applied. The impacts were classified as direct impacts, systematic impacts and wider impacts. These categories all refer to impacts that originate in automation technology, i.e. are stages of causal chains that start with technology. In addition, this technology could have secondary impacts. These impacts were modelled as behavioural adaptation and presented as a second impact diagram. The secondary impacts originate in changes in behaviour in response to the technology. The diagram showing primary impacts is shown in Figure 4, and one showing secondary impacts (behavioural adaptation; feedback) is presented in Figure 5. The impact generators in the second diagram are the direct impacts in the first diagram.

## Further steps to impact assessment

The diagrams presented in Figure 4 and Figure 5 show potential impacts and the relationship between these impacts. This first step helps create a holistic overview, but cannot be applied directly for quantitative impact assessment.

Key elements that need further development include a more detailed description of each impact presented in the diagram, specifying the direction of change of the interrelations (positive or negative), and finally identifying the mathematical forms of the relationships between impacts, i.e., estimating dose response curves, indicating how impacts depend on the market penetration rate of connectivity and automation technology.

**Figure 6.**
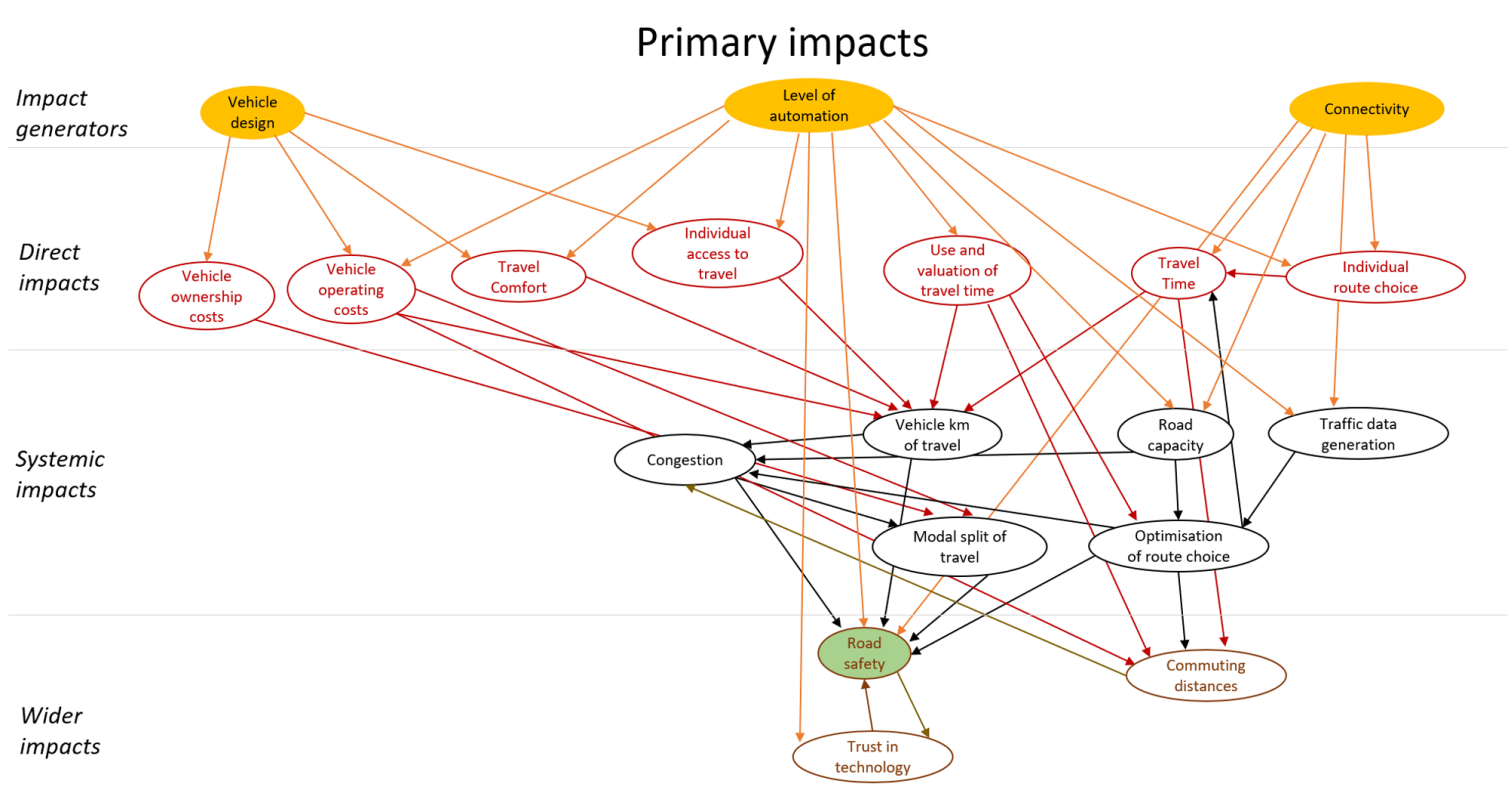
Primary impacts related to road safety.

**Figure 7.**
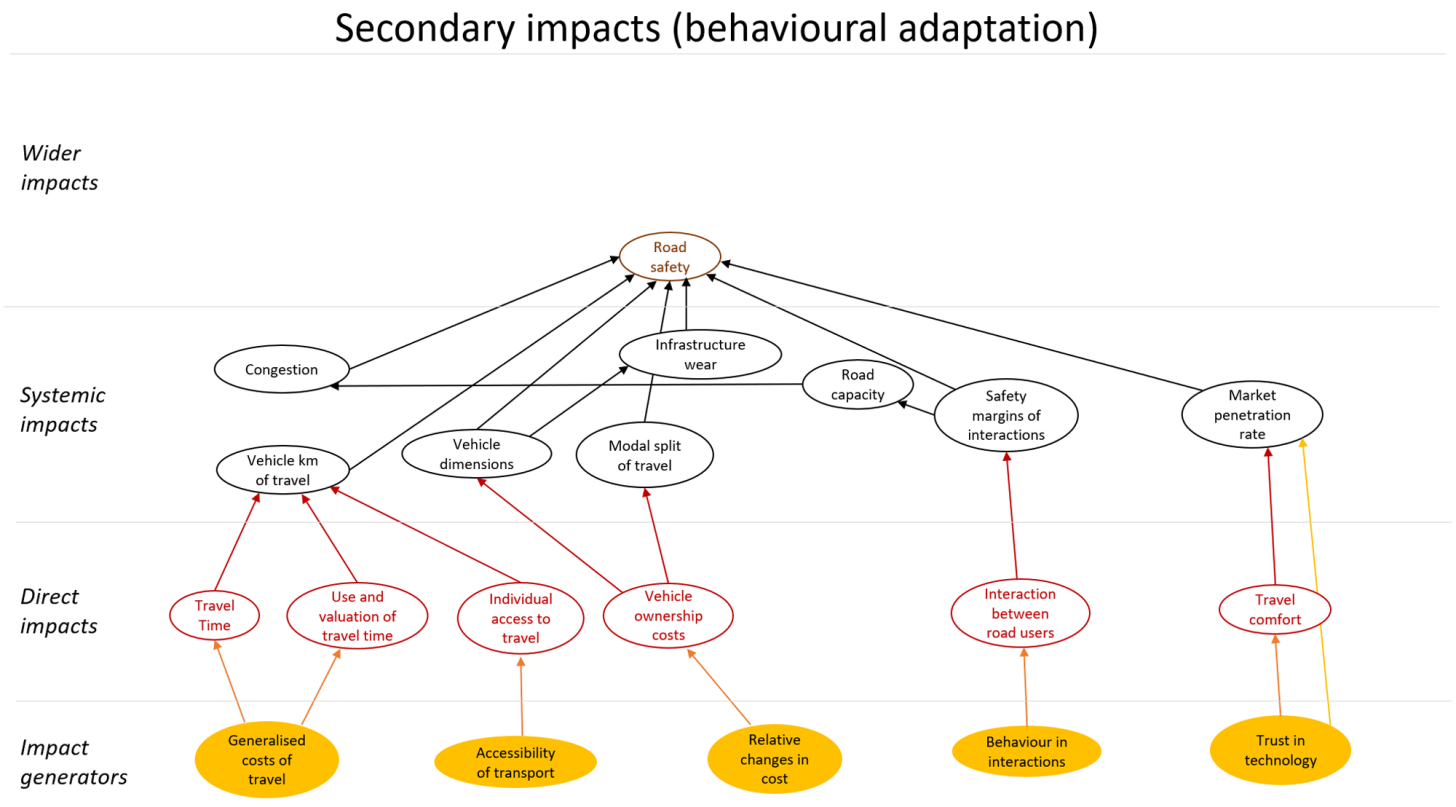
Secondary impacts related to road safety.

A first step to be taken is to limit the scope further. The impact diagram can be used to define relevant use cases based on which impacts are relevant among all those included in the diagram. Also, it is possible to focus on specific impacts. For example, one can decide to only look at safety impacts, while taking into account feedback loops caused by other types of impact that became apparent through the original broad scope diagram. In this case, an impact diagram only focusing on road safety is, for example, reduced to the primary impacts shown in Figure 6 and the secondary impacts shown in Figure 7.

Figure 6 shows that automated vehicles affect road safety directly (primary impact) via many routes, for example, they will probably have a lower risk of being involved in a crash than human driven vehicles, with the risk decreasing with increasing the level of automation. This impact is indicated in Figure 6 with the arrow between “level of automation” and “road safety”. Especially if vehicles are able to communicate with each other, i.e. if they are connected (CAVs), the risk of a crash will probably be reduced. This additional improvement on road safety is indicated with the arrow between “connectivity” and “road safety”. In addition, some potential feedback effects can be expected as shown in Figure 7. Such feedback effects can either amplify or reduce the original impact. It is, for example, likely that modal split and total distance travelled are affected by changes in generalized and relative costs of travel due to increasing levels of (C)AVs. It is known that modal split and distance travelled in turn have an impact on the number of crashes.

A logical next step in impact assessment is to quantify as many of the impacts as possible. Within the LEVITATE project this is still work in progress. One can see each interrelation as an open loop system to simplify the development of such algorithms. When doing this, potential time delays between cause and effect should also be taken into account.

## Discussion

In the LEVITATE project the presented first steps of the impact assessment method helped create a holistic overview of the impacts relevant for the further course of the project. The approach was inspired by the causal loop diagrams and methods adopted by ( Innamaa *et al.*, 2018b) and ( Milakis *et al.*, 2017). The approach combines the holistic system approach of causal loop diagrams with the structured approaches applied to impact assessment for vehicle automation.

The main difference between the approach presented here and those presented in ( Innamaa *et al.*, 2018b) and ( Milakis *et al.*, 2017) is the focus on feedback loops. This explicitly recognises the fact that new technology usually has some unintended impacts in addition to the intended impacts. This approach was strongly influenced by the focus of the project on both short and long term impacts. Especially for long term impact assessment, behavioural adaptation is of upmost importance.

It has been assumed ( Aria *et al.*, 2016; Arnaout & Arnaout, 2014; Papadoulis *et al.*, 2019), for example, that smaller time headways increase road capacity and therefore decrease congestion and travel time. This assumption, however, does not take into account the well-established fact that decreased travel time creates a feedback loop that in turn increases vehicle km travelled and may increase congestion. In a worst-case scenario, travel time is unchanged, but there are more vehicles on the road creating more pollution. 

Another difference with, for example ( Innamaa *et al.*, 2018b), is that the project scope is defined in two steps. In the first step a general scope is defined, but the final scope is defined by relying on the insights about relevant impact paths obtained from the first step of the impact assessment method described here. This choice was made to avoid limiting the impact brainstorming too early in the process. By taking many different systems into account, impacts that are not directly obvious for one type of system are still considered and might turn out to, via feedback or direct relations, significantly influence the initially considered types of impact.

Moreover, an example was given of how the impact diagram can be used to define an impact diagram that focusses on one type of impact in particular, while taking all relevant feedback loops from other types of impact into account. This approach would likely provide a more holistic view for the impact assessment of one type of impact than starting from that type of impact and expanding, as many feedback loops are often not obvious initially. Also, this approach can be used to split the work between research groups focusing on different types of impact, as is often done within large projects such as LEVITATE.

Generally, the method presented here has helped structure the impact assessment process within the LEVITATE project, greatly benefitting the efficiency of our work. Furthermore, the relatively large scope in the first phase of impact assessment has benefitted the open exploration of potential impact areas and their interrelations. This strategy of delaying the definition of the final scope resembles the double diamond method often used in design processes ( Design Council, 2015; Tschimmel, 2012). Here a phase of exploration precedes the scoping phase so that first new insides are gathered and the problem is looked at in a fresh way before the final scoping occurs. This approach to impact assessment has its limitations. While the method aims to be as holistic as possible in defining the impacts, it is not possible to know if true completeness is achieved. Aiming for completeness helps to create insight in all the different factors that are interrelated and together define impacts of CAVs. To achieve this, however, the scope of the assessment is initially kept quite large. This large scope makes it harder to be specific on the exact parameters and dose response curves needed to define each impact. After the scope has been reduced, as is proposed as a next step, many more steps will need to be taken before a quantitative impact assessment can be performed. Defining a smaller scope initially can make the overall process faster, but increases the chances of failing to identify certain relevant impacts and interrelations.

## Conclusions

This paper presents the first steps of an impact assessment method for CAVs. The focus of this method is to create a holistic overview of impacts that can also be applied for long term impact assessment. The method aims to achieve this by including all feedback loops early in the process and taking different perspectives on how impacts can be classified, as well as including a validation step to assess the holisticness of the final impact diagram.

While the authors do not claim to present the only and best way to assess impacts of CAVs, this method has proven successful for the purposes of the European project LEVITATE and can be expected to help others with similar analysis challenges.

## Data availability

### Underlying data

Zenodo: Impact assessment methodology for connected and automated vehicles. https://doi.org/10.5281/zenodo.5244506 ( Cleij *et al.*, 2021).

This project contains the following underlying data:

- *Cleijetal2021_ExplorativeLiteratureOverview.pdf* (results of the explorative literature review from the diagram set up phase)
- *Cleijetal2021_OverviewOfMostRelevantLiterature.pdf* (overview of most relevant literature used during the development of the impact diagrams described in this manuscript)
- *Cleijetal2021_IntermediateResultsOfDiagramDevelopment.pdf* (overview of the intermediate results of the development process for the impact diagrams described in this manuscript)

Data are available under the terms of the Creative Commons Attribution 4.0 International license (CC-BY 4.0).

## References

[ref-1] Aria E Olstam J Schwietering C : Investigation of Automated Vehicle Effects on Driver's Behavior and Traffic Performance. *Transp Res Proc.* 2016;15:761–770. 10.1016/j.trpro.2016.06.063

[ref-2] Arnaout GM Arnaout JP : Exploring the effects of cooperative adaptive cruise control on highway traffic flow using microscopic traffic simulation. *Transport Plan Techn.* 2014;37(2):186–199. 10.1080/03081060.2013.870791

[ref-3] Bala BK Arshad FM Noh KM : System Dynamics.Singapore: Springer Singapore, 2017.

[ref-4] Chan CY : Advancements, prospects, and impacts of automated driving systems. *Int J of Trans Sci Technol.* 2017;6(3):208–216. 10.1016/j.ijtst.2017.07.008

[ref-5] Cleij D Weijermars W Elvik R : Impact assessment methodology for connected and automated vehicles (Version 1). *Zenodo.* 2021. 10.5281/zenodo.5244506 PMC11099508 38765937

[ref-6] Design Council: Design methods for developing services. 2015. Reference Source

[ref-7] Elvik R Quddus M Papadoulis A : A taxonomy of potential impacts of connected and automated vehicles at different levels of implementation.(Deliverable D3.1 of the H2020 project LEVITATE). 2019. Reference Source

[ref-8] Fagnant DJ Kockelman K : Preparing a nation for autonomous vehicles: opportunities, barriers and policy recommendations. *Transp Res Part A Policy Pract.* 2015;77:167–181. 10.1016/j.tra.2015.04.003

[ref-9] Herrmann A Brenner W Stadler R : Autonomous Driving.Bingley, UK: Emerald Publishing Limited, 2018. 10.1108/9781787148338

[ref-10] Hibberd D Louw T Aitoniemi E : From Research Questions to Logging Requirements.(Deliverable D3.1 of the European research project L3Pilot). 2017. 10.13140/RG.2.2.14755.91680

[ref-11] Hörl S Ciari F Axhausen KW : Recent perspectives on the impact of autonomous.(Working Paper by IVT and ETH Zurich No. 10XX). 2016;1216. 10.3929/ethz-b-000121359

[ref-12] Innamaa S Smith S Barnard Y : Framework for assessing the impacts of automated driving.Paper presented at the 7th Transport Research Arena, Vienna, Austria. 2018a. Reference Source

[ref-13] Innamaa S Smith S Barnard Y : Trilateral impact assessment framework for automation in road transport.(Report by the Trilateral Working Group on Automation in Road Transportation (ART WG)). 2018b. Reference Source

[ref-14] Kockelman K Avery P Bansal P : Implications of Connected and Automated Vehicles on the Safety and Operations of Roadway Networks: A Final Report.(Report No. FHWA/TX-16/0-6849-1). 2016. Reference Source

[ref-15] Kockelman K Boyles S : Smart transport for cities & nations: the rise of self-driving & connected vehicles.USA: Createspace, 2018. Reference Source

[ref-16] Litman T : Autonomous Vehicle Implementation Predictions: Implications for Transport Planning. 2019. Reference Source

[ref-17] Milakis D van Arem B van Wee B : Policy and society related implications of automated driving: A review of literature and directions for future research. *J Intell Transport S.* 2017;21(4):324–348. 10.1080/15472450.2017.1291351

[ref-18] Papadoulis A Quddus M Imprialou M : Evaluating the safety impact of connected and autonomous vehicles on motorways. *Accid Anal Prev.* 2019;124:12–22. 10.1016/j.aap.2018.12.019 30610995

[ref-19] Polis: Road Vehicle Automation and Cities and Regions.(Discussion paper by Polis Traffic Efficiency & Mobility Working Group). 2018. Reference Source

[ref-20] Sousa N Almeida A Coutinho-Rodrigues J : Dawn of autonomous vehicles: review and challenges ahead. *P I Civil Eng-Munic.* 2018;171(1):3–14. 10.1680/jmuen.16.00063

[ref-21] Tschimmel K : Design Thinking as an effective Toolkit for Innovation. *Proceedings of the XXIII ISPIM Conference: Action for Innovation: Innovating from Experience.* 2012. 10.13140/2.1.2570.3361

[ref-22] van Nes N Duivenvoorden K : Safely towards self-driving vehicles.(R-2017-2E). 2017. Reference Source

