# First steps towards a holistic impact assessment methodology for connected and automated vehicles

**DOI:** 10.12688/openreseurope.13870.3

**Published:** 2023-09-25

**Authors:** Diane Cleij, Wendy Weijermars, Rune Elvik

**Affiliations:** 1 SWOV Institute for Road Safety Research, Bezuidenhoutseweg 62, The Hague, 2594 AW, The Netherlands; 2 Institute of Transport Economics, Gaustadalleen 21, Oslo, 0349, Norway

**Keywords:** impact assessment, automated vehicles, connected vehicles, behavioural adaptation, traffic system, societal impacts

## Abstract

Connected and automated vehicles have become more common in recent years, increasing the need to assess their societal level impacts. In this paper a methodology is presented to explore and define relevant impact areas as a starting point for quantitative impact assessment. The many interrelations between impact areas increases the complexity of obtaining a complete overview. Therefore, a structured approach is used, which shows many similarities with the modelling of causal-loop-diagrams. Feedback loops between impact areas are taken into account at an early stage and methods of literature research, project team feedback, interrelation assessment and grouping are used to produce a holistic overview of impacts. The methodology was developed and applied in the European H2020 project LEVITATE. The impact taxonomy and interrelations between impact areas resulting from this project are presented and further steps needed to perform a quantitative evaluation of the impacts are discussed.

## Introduction

Vehicle automation and connectivity has become more and more common in recent years. More and more vehicles on the roads today can take over part of the driving task, such as keeping a constant speed using cruise control or avoiding lane departures using a lane keeping system. Cars with SAE level 2 automation functions, where the driver is only required to monitor the automation, are already being sold and it is expected that conditional, high and full automation functions will become available in the (near) future. While such systems are generally expected to have the potential to increase safety and decrease congestion ( Kockelman *et al.*, 2016), the actual impacts of this technology on a societal level depend on many factors ( Kockelman *et al.*, 2016; Milakis *et al.*, 2017; Sousa *et al.*, 2018). This paper presents a method to obtain a comprehensive overview of impact areas relevant for impact assessment of connected and automated vehicle technology. 

The method described here was developed within the European horizon 2020 project LEVITATE, which aims to offer policy makers insight into the wide range of impacts that vehicle automation can have on society. The policy support tool that will be developed during this project is intended to enable a wide range of policy makers to select policy interventions and assess the impacts of automated vehicles in the short, mid and long term future under different circumstances. To serve this purpose, the first step is to gain an overview of as many of the potential impact areas of connected and automated vehicles (CAVs) as possible. As within the LEVITATE project both short term and long term impacts will be assessed, not only direct, but also indirect impacts and feedback loops that apply over longer periods of time should be included. To obtain a comprehensive overview of all impact areas, a holistic approach for defining impact areas is needed. E.g., an impact area assessment approach that, rather than focussing on specific impact areas in isolation, focusses on the whole set of impact areas and their interrelations with the goal of obtaining a complete overview of impact areas. 

Most overviews of impact areas in previous literature consist of written summaries based on literature research. They often provide a categorization of the impact areas generally defined by the authors themselves. In ( Fagnant & Kockelman, 2015) impacts are first discussed under four headings: safety, congestion and traffic operations, travel behaviour impacts and freight transport. Subsequently, they present estimates of societal and personal economic benefits based on literature findings of expected changes in vehicle miles travelled, vehicle ownership, technology cost, crash rates, congestion reduction and parking. In ( Hörl *et al.*, 2016) the impacts of vehicle automation are categorized as impacts on mobility, city planning, car industry, work organisation, user profiles, delivery of goods and price. Within each category many more specific impacts and some interrelations are mentioned. In ( Chan, 2017) benefits, i.e., positive impacts, of automated vehicles are categorized under vehicle user, transportation operation and society perspectives. Many more overviews, generally based on literature research, can be found ( Herrmann *et al.*, 2018; Kockelman & Boyles, 2018; Polis, 2018). Most of these articles and reports do not provide much insight into exactly how these overviews were obtained, other than mentioning literature research was performed.

A more structured and holistic approach was taken in ( Milakis *et al.*, 2017). The authors first developed a simplified concept which represented the possible implications of automated vehicles and identified impact areas and their respective mechanisms based on their own analytical thinking. They then performed a structured literature review on the impacts of automated vehicles. By comparing their own concept and identified impacts to those found in literature, they then identify research gaps. Their concept consists of four concentric circles showing vehicle automation technology in the centre. The first order impacts of this vehicle technology on the transport system that are directly noticed by the road users are shown around this centre, followed by the second order impacts on, for example, infrastructure and land use in the third circle band. Finally, in the fourth circle band, the wider societal impacts are shown. The model attempts to show the propagation of vehicle technology impacts from direct impacts on road users to societal impacts, giving a more coherent view of the relationship between impacts.

In contrast to the previously mentioned studies based on literature review only, the study described by Milakis *et al.* (2017) approaches the challenges of creating a holistic overview of impact areas from two distinct perspectives, i.e., the perspective based on expert knowledge in combination with analytical thinking from the authors and the perspective based on literature review. By combining these two perspectives, gaps in literature were found, implying that this method is more holistic than solely using literature review. However, their method doesn’t allow for causal relations between specific impact areas to be investigated. It is possible that relevant secondary effects caused by these interrelations are therefore omitted. Additionally, as they did not report to have iterated between the two perspectives, the holism of the results strongly depends on the initial knowledge level of the experts.

A more elaborate approach is taken in ( Innamaa *et al.*, 2018a; Innamaa *et al.*, 2018b). They define nine impact groups that are displayed on a graph of spatial resolution vs. time frame. The direct impacts, those that have a relatively clear cause-effect relationship with the primary activity or action, are those of small spatial resolution and short time frame. These impacts can usually be measured in a field test and are grouped under safety, vehicle operations, personal mobility and energy/emissions. Indirect impacts, on the other hand, are defined as resulting from these direct impacts and can often not be measured in a field test. They include impacts on network efficiency, travel behaviour, public health, infrastructure and land use and socio-economic impacts.

In a first step of the impact analysis approach described by Innamaa *et al.*, ( 2018a, 2018b) they perform a classification of the system and the design domain. In this step they, for example, make clear which automated functions and services will be included in the impact analysis. For the impact evaluation they then propose charts indicating potential impact paths starting from direct impacts on vehicle operations, driver or traveller, quality of travel and transport system and leading to one of the previously mentioned impact areas, such as safety. In addition, they recommend not only investigating these one-way paths to the impact areas, but also the strong links between the impact areas. As a next step, they recommend elaborating further on the proposed impact paths for the system under evaluation by adding direction of change, similar to what is done in causal-loop-diagrams.

In contrast to ( Milakis *et al.*, 2017), the approach described in Innamaa *et al.* ( 2018a, 2018b) does have a strong focus on interrelations between impact areas. The authors do recommend to further assess indirect impacts. Rather than the elaborate literature review presented in ( Milakis *et al.*, 2017), however, this approach starts from the impact areas defined in one overview study ( Smith *et al.*, 2015). The impact areas and interrelations were later refined at several conferences and meetings. The articles do not elaborate on how either the further assessment of indirect impacts or the mentioned refinements should be or were be performed. 

This paper presents the approach taken in LEVITATE to explore and define impact areas and their interrelations as a starting point for quantitative impact assessment. The modelling approach shows many similarities with the modelling of causal loop diagrams ( Bala *et al.*, 2017). The approach includes iterations between four distinct methods: literature research, project team feedback, interrelation assessment and grouping. Each of these methods provides a different perspective on potential missing impact areas. In addition, the scope is initially kept relatively broad as to not constrain the identification of impact areas and increase the chances of identifying relevant interrelations. The combination of an iterative approach, combining multiple perspectives and initially retaining a broad scope is expected to provide a more holistic overview of impact areas than when adopting only one of these methods and/or limiting the scope beforehand. 

In the following sections a brief, non-exhaustive list of existing literature on impact analysis of automated vehicles is discussed after which the approach developed within LEVITATE to explore the impact areas is presented. The model developed is then presented, containing both direct and indirect impact areas and their interrelations that can be easily adapted and extended for specific uses. Finally, the approach is evaluated for different uses and improvements are discussed.

## Impact assessment method

**Figure 1.**
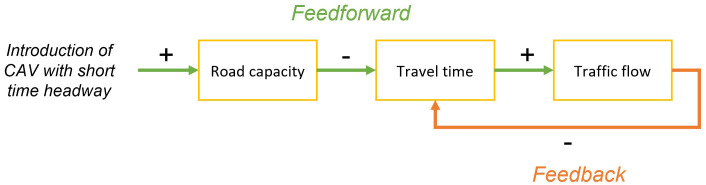
Example of causal relations between impacts, containing both feedforward (green) and feedback relations (orange).

In the LEVITATE project the focus is put on the system as a whole from the start, thus including both feedforward and feedback casual relations between different impact areas. Examples of such causal relations are shown in Figure 1. Here the feedforward, or direct, relation is the potential impact of CAV regarding the reduction of travel time due to the adoption of shorter time headways. The feedback relation is then the relation between increased traffic flow due to this shorter travel time that in turn increases the travel time.

The impact assessment method can be divided into four steps

1. Definition of scope
2. Impact area diagram set up
3. Impact area diagram elaboration
4. Impact area diagram validation

### Initial scoping

To increase the chances of identifying all relevant direct and indirect impact areas, no scoping is defined for which impact areas are included or not. Instead, only initial scoping of technologies, applications and timespans is defined. The initial definition of scope defined use cases in terms of type of technology (automation, connectivity, mobility as a service) and area of application (passenger cars, urban transport, freight transport). The LEVITATE project focuses on societal level impacts of CAVs in three areas of use: freight, urban and passenger car transport. In Table 1 the LEVITATE scope in terms of more detailed subsystems and technologies within these three areas are shown.

**Table 1.** Example connected and automated vehicles deployment scenarios for each use case.

Use case	Automated urban transport	Passenger cars	Freight transport
**Automation** ** scenarios**	• Point to point shuttle • Anywhere to anywhere shuttle • Segregated pathway operations • On road operations • Intermodal route planning • Street design implications	• SAE L2/3/4 automation • Highway pilot • Autopark • Highway pilot • Cooperative automatic cruise control • Traffic jam pilot • City chauffeur	• Highway platooning • Automated urban delivery • Depot to depot automated transfer • Automated intermodal transport • Synchronized traffic load on bridges • Intelligent access control of infrastructure/bridge
**Connectivity** ** scenarios**	• Green light optimized speed advisory • System-aware route optimization	• Geo-fencing based powertrain use • Green light optimized speed advisory • Road use pricing • System-aware route optimization	• Geo-fencing based powertrain use • Green light optimized speed advisory • Road use pricing • System-aware route optimization
**Mobility as a** ** service**	• Multi-modal integrated payments • e-hailing • Automated ride sharing	• Multi-modal integrated payments • Shared ownership models • Urban platooning	• Local freight consolidation

As the output of the LEVITATE project will be a policy support tool that can be used by municipalities, regional authorities and national governments, impacts on, for example, a European level are outside the impact assessment scope. Finally, the time periods used for the impact assessment are short (five years), medium (10 years) and long term (25+ years). These time periods correspond to the immediate introduction of mobility technologies, the duration of a mixed fleet of non-automated, partial and fully automated vehicles as well as the increase in mobility services based on increasingly ubiquitous connectivity. Within the policy support tool, impacts are estimated for different penetration rates of first and second generation automated vehicles as well as a number of additional policy measures and technologies. The tool will quantify the impacts presented in this paper accordingly.

For example, there are many vehicle-based automation technologies that are close to market. It can be assumed that these will soon enter the vehicle fleet and result in changes compared to current driving. Over the medium term there will be a mixed fleet of vehicles and a range of levels of infrastructure connectivity which may introduce new transport risks, making safety benefits uncertain. Beyond 25 years there will be largely ubiquitous automation with high levels of system integration. Cities are expected to transform as land use, employment and disruptive technologies are expected to cause unexpected changes.

### Impact area diagram set up

For setting up the impact diagram the methods of literature research and project team feedback were used. An explorative literature review on the impacts of CAVs within the scope as defined in the previous paragraph was performed. The review was done using the snowball method through Google Scholar, starting from the paper of Milakis *et al.* (2017) as this paper already describes a structured literature research on impacts of automated vehicles (last search on December 20, 2018). For each study, a list was made of the potential impact areas they identified. These lists were then compared. A consolidated list was made from all potential impact areas that were mentioned in at least one of the studies that were reviewed. An overview of the impact areas described in the found literature (see “ExplorativeLiteratureOverview.pdf” ( Cleij *et al.*, 2021)) was sent to other members of the project and their feedback was requested. The project member feedback based on their respective perspectives (research, policy making, stakeholder) and expertise (mobility, road safety, environmental sciences, systems engineering, social sciences, economics) was used to update the list of potential impact areas from literature.

To visualise the impact areas and their interrelations, the impact areas were placed in text balloons and the interrelations between these areas visualized using arrows. The arrowhead indicates the direction of the impact relation, i.e., that changes in travel time will likely impact the commuting distance is indicated with an arrow from the former towards the latter.

To structure the diagram and define an initial set of starting points generating these impacts, the top of the diagram contains the technological changes that drive the impacts; the impact generators. In the LEVITATE project the following impact generators were defined after some iterations: vehicle design, level of automation and connectivity. All impacts could be derived from these impact generators.

**Figure 2.**
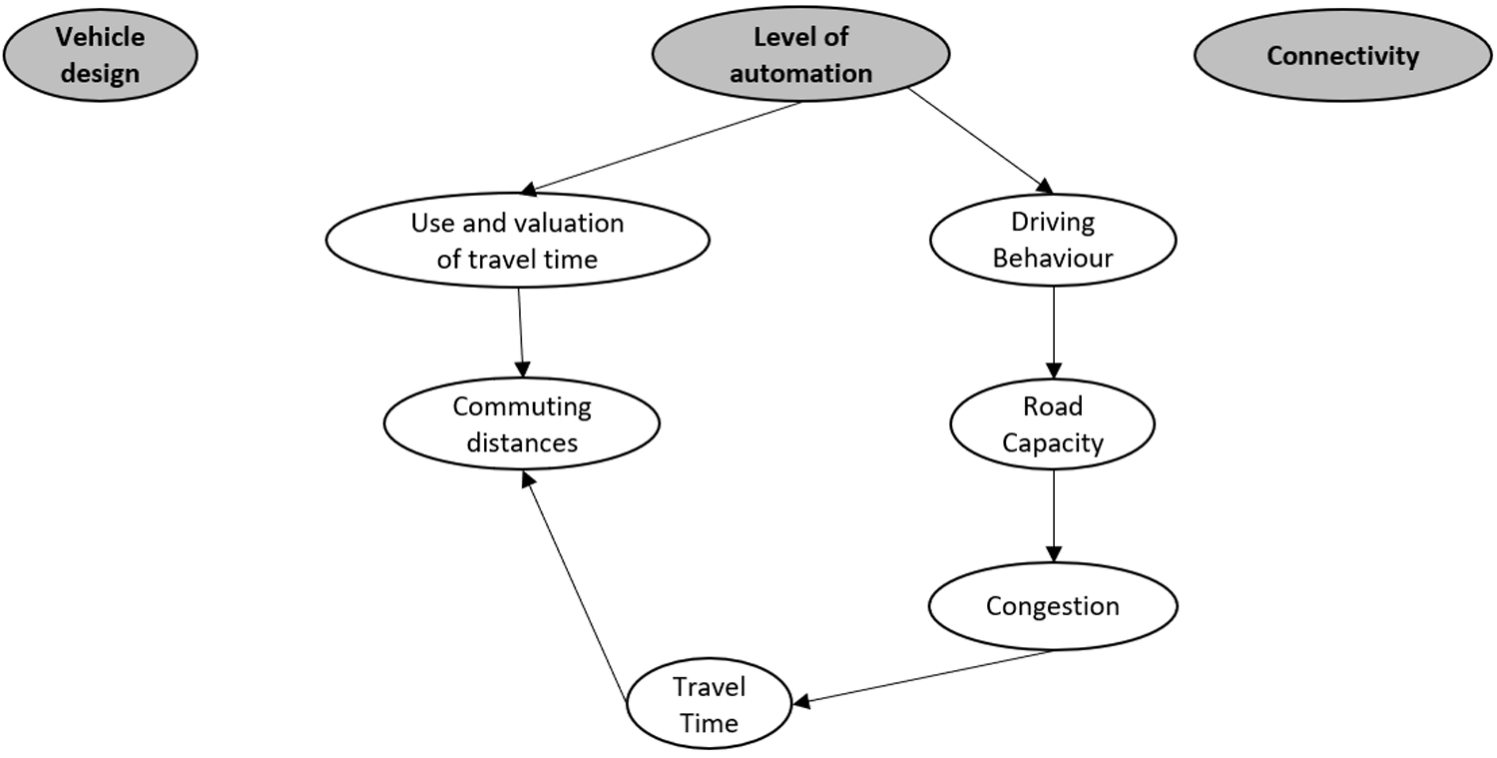
Example of impact area diagram set up with three technology areas as impact generators and six possible impact areas and their interrelations.

A simplified example of such an initial impact area diagram set up including only six impact areas is shown in Figure 2. This example shows the influence of automation level on the use and valuation of travel time and the driving behaviour (e.g., shorter headways). These in turn influence the commuting distances and road capacity, respectively. The road capacity in turn influenced the congestion, which influences travel time. Travel time in turn, influences commuting distances.

### Impact area diagram elaboration

To extend and improve the initial impact area diagram, two main methods were adopted: interrelation assessment and grouping. During the interrelation assessment , each impact area in the diagram was analyzed for possible further relations to other impact areas in the diagram and impact areas not yet in the diagram. In Figure 2, for example, no interrelations between two of the impact generators was found and this could therefore be a first clue that impact areas related to these impact generators are missing. In search for such additional impact areas, additional literature was often consulted. An overview of the most relevant literature used for the development of the impact area diagrams can also be found in the underlying data document “OverviewOfMostRelevantLiterature.pdf” ( Cleij *et al.*, 2021).

The resulting impact area diagrams can become quite complex, containing a large amount of identified impact areas and interrelations. A second, step is therefore performed to create a clearer overview of the impact areas and identify which group of impact areas has potentially not been sufficiently explored. In this step the impact areas are grouped along dimensions commonly found in the literature. The choice for such dimensions was based on a comparison of impact taxonomies from literature (see Table 2).

**Table 2.** Connected and automated vehicle impact taxonomies from literature.

( Chan, 2017)		( Milakis *et al.*, 2017)	( Polis, 2018)	( Innamaa *et al.*, 2018b)	( Hibberd *et al.*, 2017)
*Main groups*	*subgroups*				
**Vehicle users**	Comfort Convenience Mobility	Travel costs Vehicle ownership and sharing Travel choices Location choices	Travel behaviour	Travel behaviour Personal mobility	Mobility
**Transport** ** operations**		Road capacity Transport infrastructure	Spatial aspects Infrastructure Traffic efficiency	Land use Network efficiency Infrastructure Vehicle operations	Efficiency
**Society**	Environment Energy Economy Safety	Land use Energy consumption Safety Social equity Economy Public health	Road safety Socio-economic	Socio-economic Safety Energy/emissions Public health	Socio-economic Safety Environment

**Figure 3.**
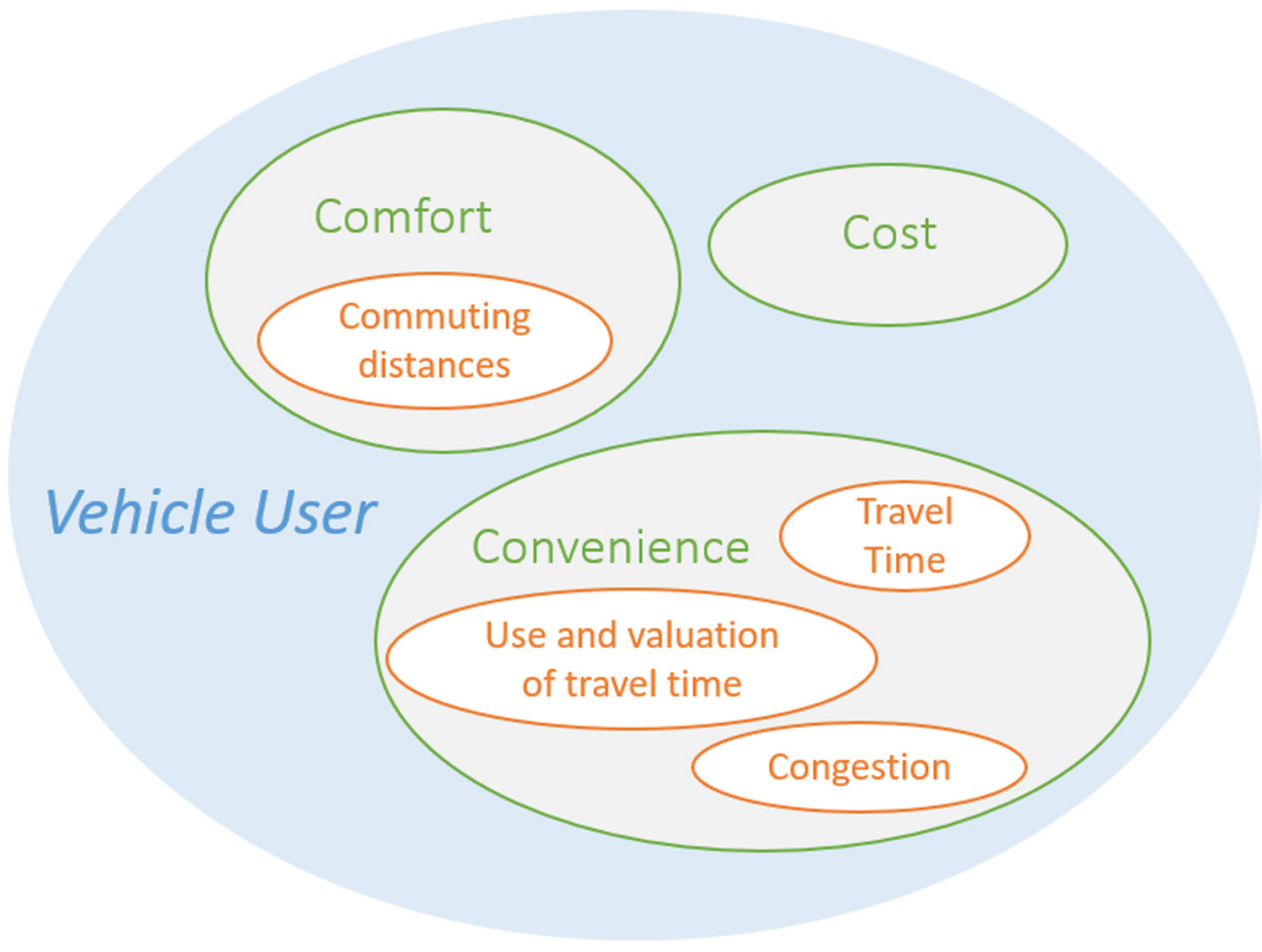
Example of impact area grouping.

The main groups in the taxonomy described in ( Chan, 2017) was deemed most holistic as it encompassed all others. The impact areas identified were therefore classified accordingly, i.e., affecting vehicle users (direct), transportation operations (systemic) and society (wider). In Figure 3 an example is given of such grouping for the impact areas from Figure 2 that can be placed in the vehicle user group. It is possible that impact areas can logically be placed in multiple groups. Commuting distances, for example, can affect both convenience and comfort. One way to address this is to duplicate this impact area and place it in both groups. Alternatively, the impact area can be placed in the group that contains the least impact areas. Here we chose the latter, as the closely related impact area of travel time was already present in the group “Convenience” and duplication would increase the complexity of the overview. To extend the impact diagram, each of these subgroups was analyzed for missing impact areas and newly found impacts were added to the overall impact area diagram. Figure 2, for example, implies that the groups “Cost” and “Comfort” are potentially not sufficiently explored and require further attention. Methods of literature research or project team feedback with a focus on impacts related to these groups can be adopted to identify additional impact areas within these groups.

The steps described in the presented approach focus on the analysis of impacts from different perspectives, which, as also shown in Milakis *et al*. (2017), increases the chance of identifying missing impact areas. Iterating over these two steps further increases this chance, making the resulting impact area diagram more holistic.

### Impact diagram validation

After several iterations of the impact area diagram elaboration step, a final impact area diagram was obtained. Whether the diagram includes all potential impact areas of CAVs cannot be ascertained at this time. However, the completeness of the diagram is an important objective of the LEVITATE project. Therefore, a validation of the completeness of the diagram was approximated by comparing the impact area diagrams to impact areas found in additional literature, in combination with a final review by project members. The literature used for this validation ( Litman, 2019; Sousa *et al.*, 2018; van Nes & Duivenvoorden, 2017) was not part of the initial explorative literature review. No additional impact areas or interrelations were found and therefore the completeness of the diagram was deemed sufficiently validated.

### Ethics statement

The consultations within this work were performed by other members of the LEVITATE project. Following the grant agreement, these project members consented to use their views.

## Method output: impact model

**Figure 4.**
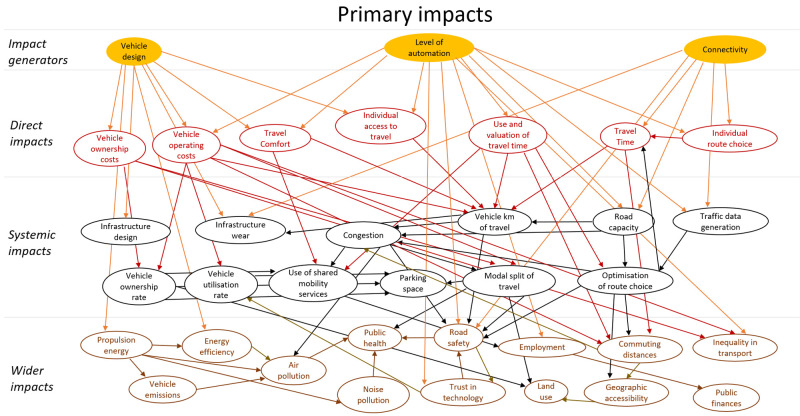
Impact area diagram with primary impacts from ( Elvik *et al.*, 2019).

**Figure 5.**
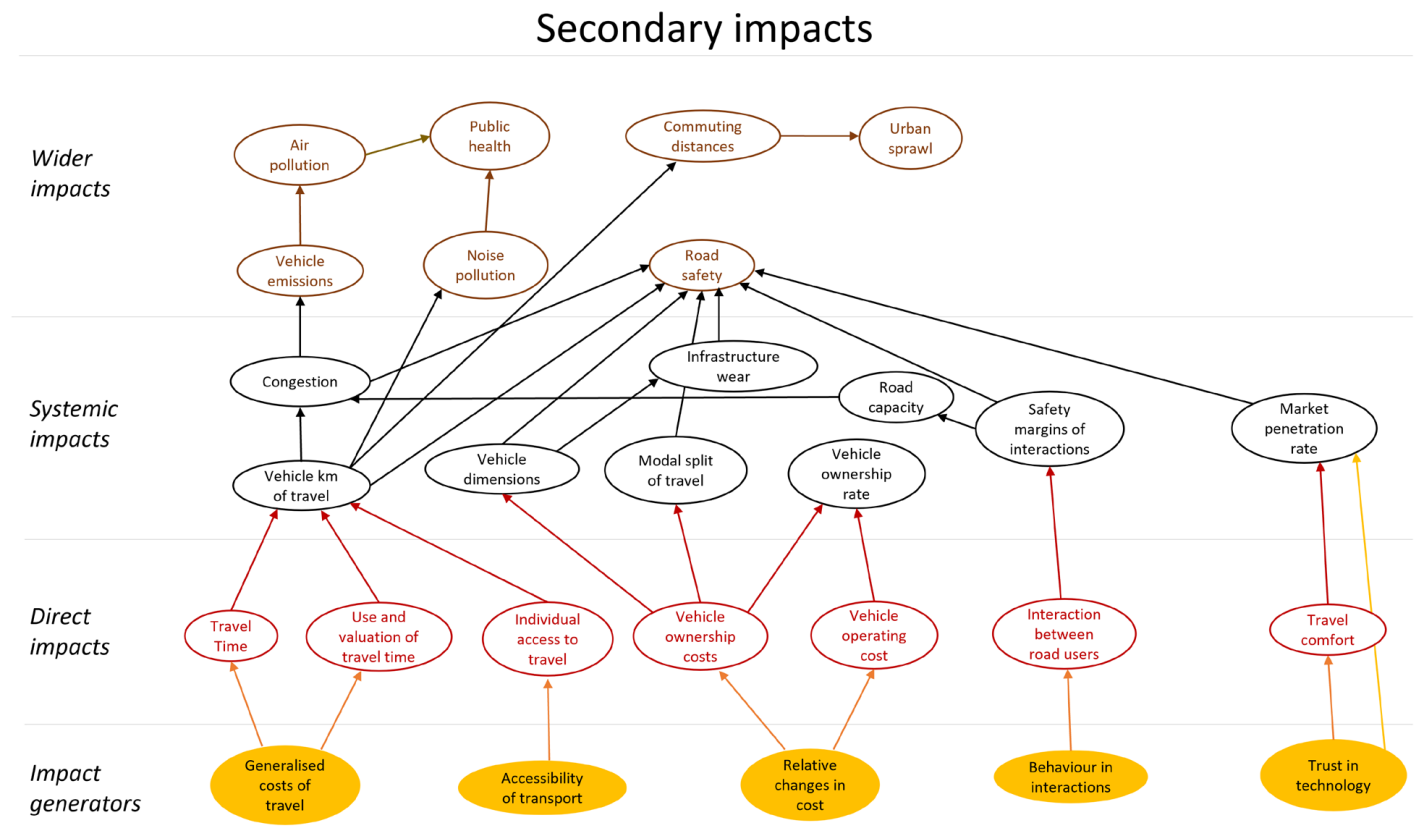
Impact area diagram with secondary impacts from ( Elvik *et al.*, 2019).

The final model of impact areas is a large complex diagram. To add structure to the diagram a similar approach to the model presented in ( Milakis *et al.*, 2017) was applied. The impact areas were classified as direct impacts, systematic impacts and wider impacts. These categories all refer to impact areas that originate in automation technology, i.e. are stages of causal chains that start with technology. In addition, this technology could have secondary impacts. These impacts were modelled as behavioural adaptation and presented as a second impact area diagram. The secondary impacts originate in changes in behaviour in response to the technology. The diagram showing areas of primary impacts is shown in Figure 4, and one showing areas of secondary impacts (behavioural adaptation; feedback) is presented in Figure 5.

## Further steps to impact assessment

The diagrams presented in Figure 4 and Figure 5 show potential impact areas and the relationship between these impact areas. This first step helps create a holistic overview, but cannot be applied directly for quantitative impact assessment.

Key elements that need further development include a more detailed description of each impact area presented in the diagram in terms of the actual impact, i.e., specifying the direction of change of the interrelations (positive or negative), and identifying the mathematical forms of the relationships between impacts, i.e., estimating dose response curves, indicating how impacts depend on the market penetration rate of connectivity and automation technology.

**Figure 6.**
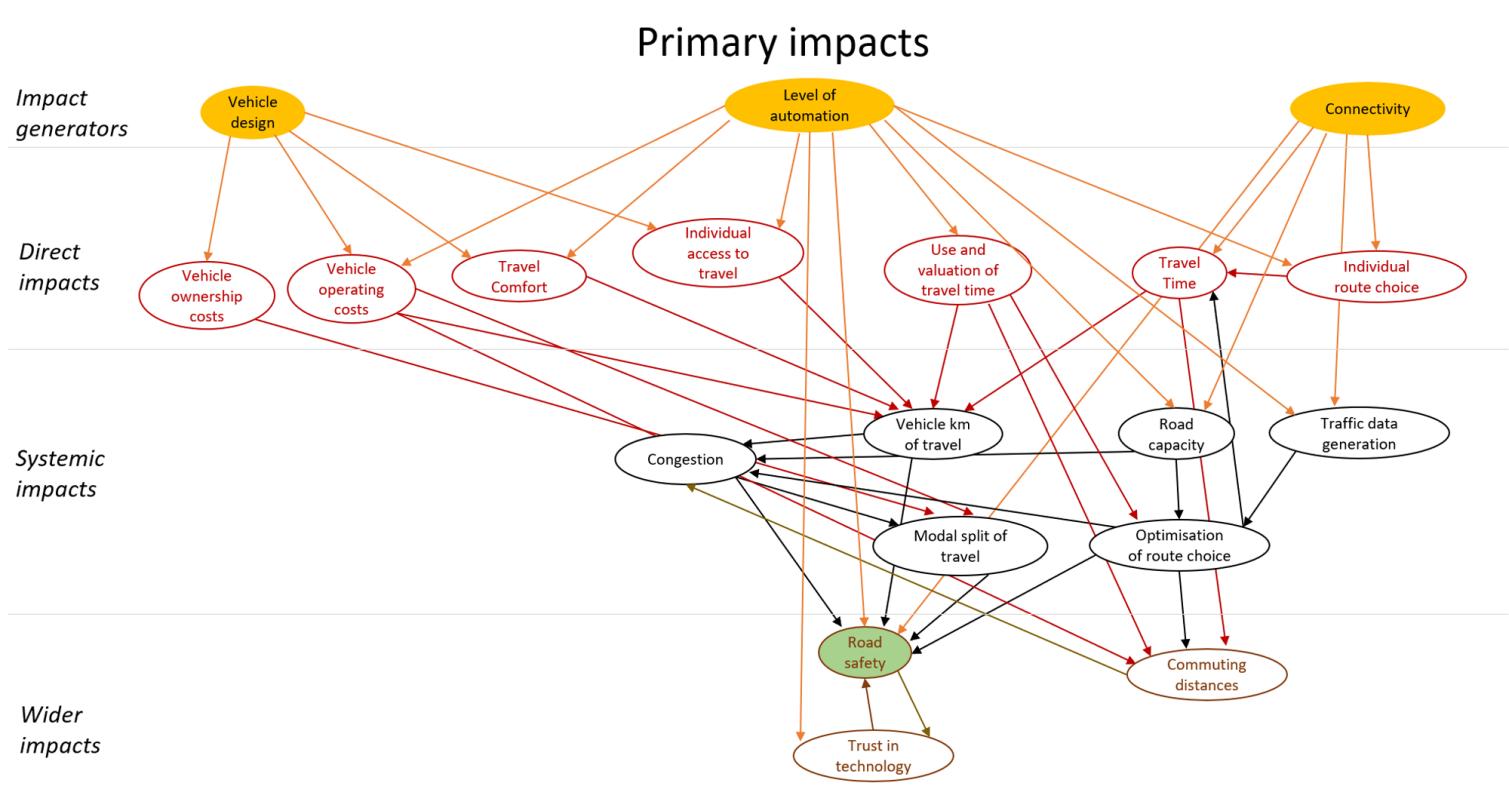
Primary impact areas related to road safety.

**Figure 7.**
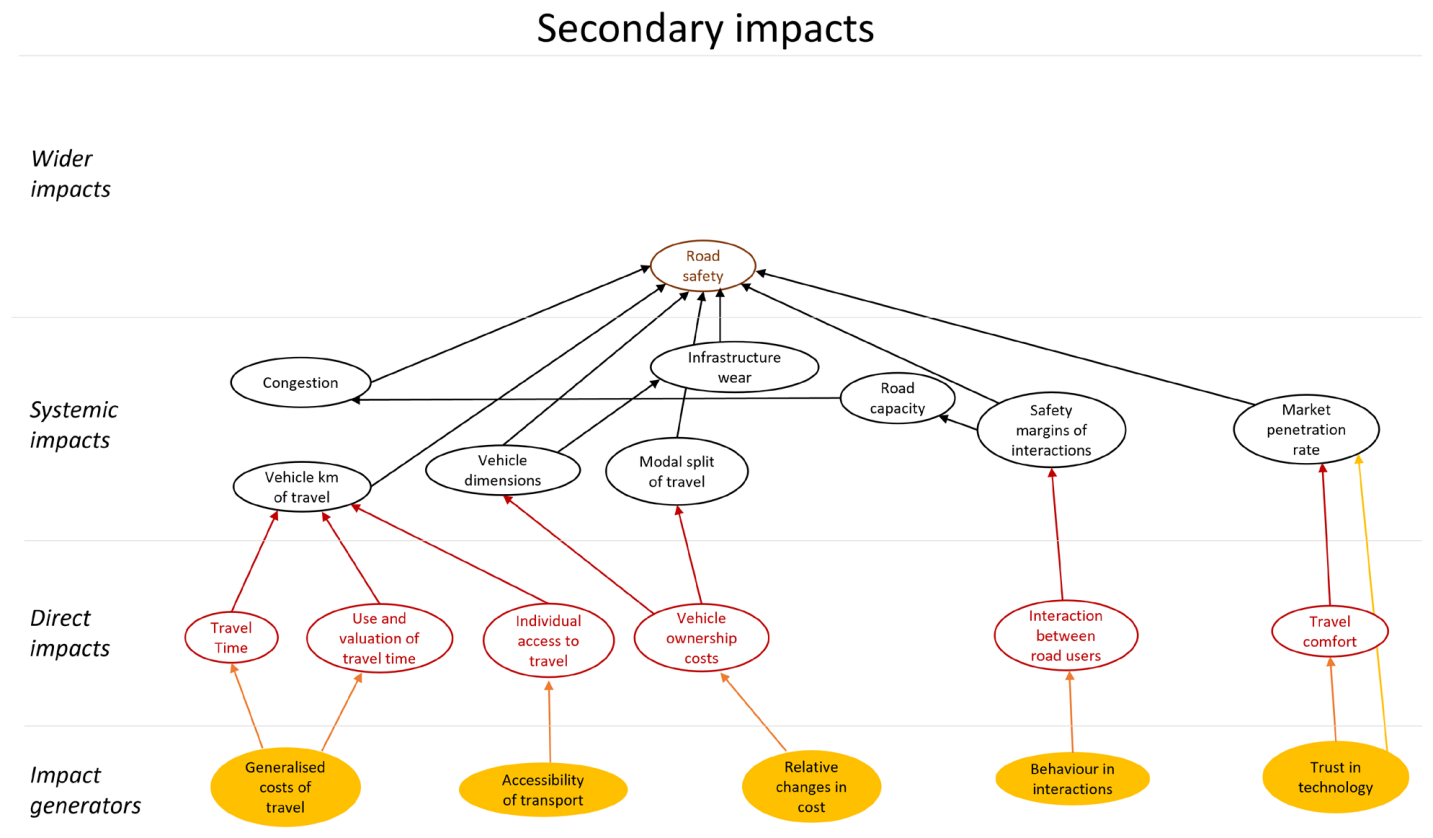
Secondary impact areas related to road safety.

A first step to be taken is to limit the scope further. , One way of doing so is by focussing only on specific impact areas. For example, one can decide to only look at safety impacts, while taking into account feedback loops caused by other types of impact that became apparent through the original broad scope diagram. In this case, an impact area diagram only focusing on road safety is, for example, reduced to the areas of primary impacts shown in Figure 6 and the areas of secondary impacts shown in Figure 7.

Figure 6 shows that automated vehicles affect road safety directly (primary impact) via many routes, for example, as automated vehicles have different capabilities and limitations as compared to human driven vehicles, they will likely also have a different risk of being involved in a crash than human driven vehicles. The risk changes likely increase with increasing level of automation, as the human involvement decreases. This impact is indicated in Figure 6 with the arrow between “level of automation” and “road safety”. If vehicles are able to communicate with each other, i.e. if they are connected (CAVs), the risk of a crash will also be affected. This additional change to road safety is indicated with the arrow between “connectivity” and “road safety”. In addition, some potential feedback effects can be expected as shown in Figure 7. Such feedback effects can either amplify or reduce the original impact. It is, for example, likely that modal split and total distance travelled are affected by changes in generalized and relative costs of travel due to increasing levels of IAVs. It is known that modal split and distance travelled in turn have an impact on the number of crashes.

A logical next step in impact assessment is to quantify as many of the impacts as possible. Within the LEVITATE project this is still work in progress. One can see each interrelation as an open loop system to simplify the development of such algorithms. When doing this, potential time delays between cause and effect should also be taken into account.

Within the LEVITATE project, the focus was not only on forecasting impacts of automated vehicles, but also on backcasting, i.e., identifying policy measures that would result in desired impacts within a set time span. Within LEVITATE the impact area diagram was also used to brainstorm about relevant policy measures by providing details on interrelations between the desired areas of wider societal impact and the areas of the lower level direct impacts. For example, if a desired societal impact is reducing air pollution, the interrelations in Figure 4 imply that one can, amongst others, develop policy interventions to influence the “vehicle design” (which in turn influences the “energy efficiency”) or to influence “connectivity” (which in turn has an effect on “road capacity” and subsequently on “congestion”). Based on such analyses a set of relevant policy interventions for further detailed analysis can be defined. 

## Discussion

In the LEVITATE project the presented first steps of the impact assessment method helped create a holistic overview of the impact areas relevant for the further course of the project. The approach was inspired by the causal loop diagrams and methods adopted by ( Innamaa *et al.*, 2018b) and ( Milakis *et al.*, 2017). The approach presented here combines four distinct methods: literature research, project team feedback, interrelation assessment and grouping. The scope is initially kept relatively broad as to not constrain the identification of impact areas and increase the chances of identifying relevant interrelations. 

The main difference between the approach presented here and those presented in ( Innamaa *et al.*, 2018b) and ( Milakis *et al.*, 2017) is the combination of looking at the impact areas from different perspectives by adopting multiple methods and iterating through these methods to minimize the omission of impact areas and with that obtain a holistic overview of impact areas. 

Another relevant difference is the strong focus on feedback loops. This explicitly recognises the fact that new technology usually has some unintended impacts in addition to the intended impacts. This approach was strongly influenced by the focus of the project on both short and long term impacts. Especially for long term impact assessment, behavioural adaptation is of upmost importance.

It has been assumed ( Aria *et al.*, 2016; Arnaout & Arnaout, 2014; Papadoulis *et al.*, 2019), for example, that smaller time headways increase road capacity and therefore decrease congestion and travel time. This assumption, however, does not take into account the well-established fact that decreased travel time creates a feedback loop that in turn increases vehicle km travelled and may increase congestion. In a worst-case scenario, travel time is unchanged, but there are more vehicles on the road creating more pollution. 

Another difference with, for example ( Innamaa *et al.*, 2018b), is that the project scope is defined in two steps. In the first step a general scope of the technologies, applications and timespans that will be addressed is defined, but no scoping related to relevant impact areas is made. The final scope regarding technologies, applications and impact areas was defined at a later stage in the project by relying on the insights about relevant impact paths obtained from the first step of the impact assessment method described here. This choice was made to avoid limiting the impact brainstorming too early in the process. By taking many different systems and impact areas into account, impact areas that are not directly obvious for one type of system are still considered and might turn out to, via feedback or direct relations, significantly influence the initially considered types of impact.

Moreover, an example was given of how the impact area diagram can be used to define an impact area diagram that focusses on one type of impact in particular, while taking all relevant feedback loops from other types of impact into account. This approach would likely provide a more holistic view for the impact assessment of one type of impact than starting from that type of impact and expanding, as many feedback loops are often not obvious initially. Also, this approach can be used to split the work between research groups focusing on different types of impact, as is often done within large projects such as LEVITATE.

Generally, the method presented here has helped structure the impact assessment process within the LEVITATE project, greatly benefitting the efficiency of our work. Furthermore, the relatively large scope in the first phase of impact assessment in combination with the adoption of several different methods to identify impact areas has benefitted the open exploration of potential impact areas and their interrelations. The strategy of delaying the definition of the final scope resembles the double diamond method often used in design processes ( Design Council, 2015; Tschimmel, 2012). Here a phase of exploration precedes the scoping phase so that first new insides are gathered and the problem is looked at in a fresh way before the final scoping occurs. 

This approach to impact assessment does have its limitations. While the method aims to be as holistic as possible in defining the impact areas, it is not possible to know if true completeness is achieved. It is therefore advised to continue to regularly revisit and update the diagram if necessary. 

Aiming for completeness helps to create insight in all the different factors that are interrelated and together define impact areas of CAVs. To achieve this, however, the scope of the assessment is initially kept quite large. This large scope makes it harder to be specific on the exact parameters and dose response curves needed to define each impact. After the scope has been reduced, as is proposed as a next step, many more steps will need to be taken before a quantitative impact assessment can be performed. Defining a smaller scope initially can make the overall process faster, but Increases the chances of failing to identify certain relevant impacts and interrelations.

## Conclusions

This paper presents the first steps of an impact assessment method for CAVs. The focus of this method is to create a holistic overview of impact areas that can also be applied for long term impact assessment. The method aims to achieve this by including all feedback loops early in the process and taking different perspectives on how impact areas can be classified, as well as including a validation step to assess the holisticness of the final impact area diagram.

While the authors do not claim to present the only and best way to assess impacts of CAVs, this method has proven successful for the purposes of the European project LEVITATE and can be expected to help others with similar analysis challenges.

## Data Availability

Zenodo: Impact assessment methodology for connected and automated vehicles. https://doi.org/10.5281/zenodo.5244506 ( Cleij *et al.*, 2021). This project contains the following underlying data: *Cleijetal2021_ExplorativeLiteratureOverview.pdf* (results of the explorative literature review from the diagram set up phase) *Cleijetal2021_OverviewOfMostRelevantLiterature.pdf* (overview of most relevant literature used during the development of the impact diagrams described in this manuscript) *Cleijetal2021_IntermediateResultsOfDiagramDevelopment.pdf* (overview of the intermediate results of the development process for the impact diagrams described in this manuscript) Data are available under the terms of the Creative Commons Attribution 4.0 International license (CC-BY 4.0).

## References

[ref-1] Aria E Olstam J Schwietering C : Investigation of Automated Vehicle Effects on Driver's Behavior and Traffic Performance. *Transp Res Proc.* 2016;15:761–770. 10.1016/j.trpro.2016.06.063

[ref-2] Arnaout GM Arnaout JP : Exploring the effects of cooperative adaptive cruise control on highway traffic flow using microscopic traffic simulation. *Transport Plan Techn.* 2014;37(2):186–199. 10.1080/03081060.2013.870791

[ref-3] Bala BK Arshad FM Noh KM : System Dynamics.Singapore: Springer Singapore, 2017.

[ref-4] Chan CY : Advancements, prospects, and impacts of automated driving systems. *Int J of Trans Sci Technol.* 2017;6(3):208–216. 10.1016/j.ijtst.2017.07.008

[ref-5] Cleij D Weijermars W Elvik R : Impact assessment methodology for connected and automated vehicles (Version 1). *Zenodo.* 2021. 10.5281/zenodo.5244506 PMC11099508 38765937

[ref-6] Design Council: Design methods for developing services. 2015. Reference Source

[ref-7] Elvik R Quddus M Papadoulis A : A taxonomy of potential impacts of connected and automated vehicles at different levels of implementation.(Deliverable D3.1 of the H2020 project LEVITATE). 2019. Reference Source

[ref-8] Fagnant DJ Kockelman K : Preparing a nation for autonomous vehicles: opportunities, barriers and policy recommendations. *Transp Res Part A Policy Pract.* 2015;77:167–181. 10.1016/j.tra.2015.04.003

[ref-9] Herrmann A Brenner W Stadler R : Autonomous Driving.Bingley, UK: Emerald Publishing Limited, 2018. 10.1108/9781787148338

[ref-10] Hibberd D Louw T Aitoniemi E : From Research Questions to Logging Requirements.(Deliverable D3.1 of the European research project L3Pilot). 2017. 10.13140/RG.2.2.14755.91680

[ref-11] Hörl S Ciari F Axhausen KW : Recent perspectives on the impact of autonomous.(Working Paper by IVT and ETH Zurich No. 10XX). 2016;1216. 10.3929/ethz-b-000121359

[ref-12] Innamaa S Smith S Barnard Y : Framework for assessing the impacts of automated driving.Paper presented at the 7th Transport Research Arena, Vienna, Austria. 2018a. Reference Source

[ref-13] Innamaa S Smith S Barnard Y : Trilateral impact assessment framework for automation in road transport.(Report by the Trilateral Working Group on Automation in Road Transportation (ART WG)). 2018b. Reference Source

[ref-14] Kockelman K Avery P Bansal P : Implications of Connected and Automated Vehicles on the Safety and Operations of Roadway Networks: A Final Report.(Report No. FHWA/TX-16/0-6849-1). 2016. Reference Source

[ref-15] Kockelman K Boyles S : Smart transport for cities & nations: the rise of self-driving & connected vehicles.USA: Createspace, 2018. Reference Source

[ref-16] Litman T : Autonomous Vehicle Implementation Predictions: Implications for Transport Planning. 2019. Reference Source

[ref-17] Milakis D van Arem B van Wee B : Policy and society related implications of automated driving: A review of literature and directions for future research. *J Intell Transport S.* 2017;21(4):324–348. 10.1080/15472450.2017.1291351

[ref-18] Papadoulis A Quddus M Imprialou M : Evaluating the safety impact of connected and autonomous vehicles on motorways. *Accid Anal Prev.* 2019;124:12–22. 10.1016/j.aap.2018.12.019 30610995

[ref-19] Polis: Road Vehicle Automation and Cities and Regions.(Discussion paper by Polis Traffic Efficiency & Mobility Working Group). 2018. Reference Source

[ref-23] Smith S Bellone J Bransfield SJ : Benefits Estimation Framework for Automated Vehicle Operations.2015. Reference Source

[ref-20] Sousa N Almeida A Coutinho-Rodrigues J : Dawn of autonomous vehicles: review and challenges ahead. *P I Civil Eng-Munic.* 2018;171(1):3–14. 10.1680/jmuen.16.00063

[ref-21] Tschimmel K : Design Thinking as an effective Toolkit for Innovation. *Proceedings of the XXIII ISPIM Conference: Action for Innovation: Innovating from Experience.* 2012. 10.13140/2.1.2570.3361

[ref-22] van Nes N Duivenvoorden K : Safely towards self-driving vehicles.(R-2017-2E). 2017. Reference Source

