# Supplementary material for: First steps towards a holistic impact assessment methodology for connected and automated vehicles
Source: Open Res Eur. 2023 Sep 25;1:104. Originally published 2021 Sep 7. [Version 3] doi: 10.12688/openreseurope.13870.3 (PMC11099508; doi:10.12688/openreseurope.13870.3)
Supplement: Peer review history

# Reviewer report: Reviewer response for version 3

**DOI:** 10.21956/openreseurope.17982.r39876

**Published:** 2024-06-08

**Authors:** Fahimeh Golbabaei

**Affiliations:** 1 Engineering, Queensland University of Technology, Brisbane, Queensland, Australia

This paper appears to make a significant contribution to the field of transportation research by developing a new methodological framework to analyse the complex societal impacts of emerging automotive technologies and is worth indexing.

 Please review the following for my general remarks and suggestions:

 - It is recommended to include the contribution of the research at the end of the abstract.

 - Adding these references to the Introduction section would be helpful:Refer Ref [1] [2].

 - it is suggested to improve the quality of the conclusion section. Adding another section at the end of the paper after the conclusion which discusses the limitation of the current study as well as presents some suggestions for future research is highly recommended.

Is the rationale for developing the new method (or application) clearly explained?

Partly

Is the description of the method technically sound?

Partly

Are the conclusions about the method and its performance adequately supported by the findings presented in the article?

Partly

If any results are presented, are all the source data underlying the results available to ensure full reproducibility?

No source data required

Are sufficient details provided to allow replication of the method development and its use by others?

Yes

Reviewer Expertise:

Sustainable Transport, Accessible Public Transport, Autonomous Vehicles

I confirm that I have read this submission and believe that I have an appropriate level of expertise to confirm that it is of an acceptable scientific standard, however I have significant reservations, as outlined above.

## References

[1] : The role of shared autonomous vehicle systems in delivering smart urban mobility: A systematic review of the literature. *International Journal of Sustainable Transportation* .2021;15(10) : 10.1080/15568318.2020.1798571 731-748 10.1080/15568318.2020.1798571

[2] : Individual Predictors of Autonomous Vehicle Public Acceptance and Intention to Use: A Systematic Review of the Literature. *Journal of Open Innovation: Technology, Market, and Complexity* .2020;6(4) : 10.3390/joitmc6040106 10.3390/joitmc6040106

# Reviewer report: Reviewer response for version 3

**DOI:** 10.21956/openreseurope.17982.r38872

**Published:** 2024-05-29

**Authors:** Pierre-Antoine Laharotte, Hugues Blache

**Affiliations:** 1 Université Gustave Eiffel, Lyon, France; 2 Universite Gustave Eiffel, Marne-la-Vallée, Île-de-France, France

- **Review by Dr. PA Laharotte**

This paper develops the logic to achieve a comprehensive methodology coping with the following challenges: (i) delimitation and identification of the set (universe) of "impacts areas" related to Connected and Automated Vehicles (CAV) and (ii) formalizing and depicting the design of the relationships between impact areas and factors. The authors describe the logic at stake to achieve a formal methodology for a systematic CAV impact assessment.

 The paper is well-written, clear and easy to understand.

 The previous remarks have been integrated into the revised version of the paper.

 Here are some minor complementary remarks that might help the reading of the paper:

- page 4 col. 2 "In the following sections ...": The literature review has already been introduced previously, please remove it from the description of the subsequent sections.
- page 4 col. 2 fig. 1: why is the feedback with a minus "-" for travel time? According to the previous uses of "+" and "-", it should involve an increase in travel time, i.e. "+", isn't it?
- page 5 col. 2: The "impact area diagram set up" sections might be split into two sections: "step 1: identification of the set of impact areas through a snowball-based literature review" and "Step 2: Modelling process to design the relationships between impact areas". Then, the section "Impact area diagram elaboration" would become "step 3: Implementation (Feeding) of the modelling strategy", etc. Furthermore, before introducing the steps of the methodology in detail, it would be helpful to introduce it shortly previously with items, for instance.
- page 10 col. 1 "A logical next step [...] is to quantify [...]": It would be nice to provide suggestions (have a short discussion) regarding the potential methods to assign values (positive or negative impacts) to the edges connecting "impact areas". Impact quantification is indeed the following and challenging step: an opening on the topic would support the relevance of the modelling previously introduced by the authors.

Minor revisions:

- p4 col. 2: typo mistake: "casual" instead of "causal".
- p8 col. 2: Remove the comma before "one way of doing it".

 

- **Review by Hugues Blache**

The article titled "First Steps Towards a Holistic Impact Assessment Methodology for Connected and Automated Vehicles" seems to have addressed the corrections suggested by the reviewers. It reads smoothly and provides a comprehensive view of a holistic approach within the LEVITATE project regarding the study of impacts from connected and automated vehicles across different spatial and temporal scales. We can appreciate the holistic aspect of this approach because the authors strive to also consider the indirect impacts of implementing CAV, along with societal viewpoints and political issues, even though the study only superficially touches on this complex problem The various temporal scales (5 years, 10 years, and 15 years) also fit within this holistic perspective.

 The literature review is well-executed and illustrates the current research problem, highlighting the partial or fragmented focus on impacts of connected and automated vehicles (such as solely on safety, efficiency, etc.). Through the literature review, the authors have presented their approaches using a combination of four distinct methods: literature review, project experience feedback, interrelation assessment and grouping to identify any missing impacts.

 This article illustrates clearly the different impacts of CAV and their relationships between the impact. In summary, the article provides a good perspective on the impacts of CAV on society and offers reflective tools beyond technology, particularly concerning indirect impacts.

 However, I have a few suggestions for improving the article:

- Introduction: "The actual impact of this technology on a societal level depend on many factors." Although some answers are provided in the following paragraphs, it would be wise to provide two or three examples of these factors, or rephrase this sentence.
- Table I: Authors mention *Automated Scenarios* and *Connected Scenarios*. However, it seems that these represent more systems or use cases (e.g. GLOSA). It is important to be precise in the definitions as they could be interpreted based on notions from the certification of CAV using scenario-based approaches. Refer to the article: *Ulbrich, Simon, et al. "Definition and justification of terms scene, situation, and scenario for automated driving." 2015 IEEE 18th international conference on intelligent transportation systems. IEEE, 2015.* This also raises the question about Operational Design Domains (ODD-, perhaps to be mentioned in the discussion on deployment limits of certain systems).
- Figure 5: In my opinion, road safety might have a more or less direct impact on public health.

Is the rationale for developing the new method (or application) clearly explained?

Yes

Is the description of the method technically sound?

Yes

Are the conclusions about the method and its performance adequately supported by the findings presented in the article?

Yes

If any results are presented, are all the source data underlying the results available to ensure full reproducibility?

Yes

Are sufficient details provided to allow replication of the method development and its use by others?

Yes

Reviewer Expertise:

Transport engineering – data science – machine learning – connected and automated vehicles – impact assessment

We confirm that we have read this submission and believe that we have an appropriate level of expertise to confirm that it is of an acceptable scientific standard, however we have significant reservations, as outlined above.

# Reviewer report: Reviewer response for version 3

**DOI:** 10.21956/openreseurope.17982.r39869

**Published:** 2024-05-16

**Authors:** Pavlos Tafidis

**Affiliations:** 1 The School of Engineering, The University of Edinburgh, Edinburgh, Scotland, UK

The article presents a comprehensive approach to assessing the societal impacts of connected and automated vehicles (CAVs). This methodology, developed within the European H2020 project LEVITATE, aims to provide policymakers with a holistic view of the potential impacts of CAV technology. The methodology combines literature research, expert feedback, interrelation assessment, and grouping to identify and map the direct and indirect impacts of CAVs. The paper emphasizes the importance of considering feedback loops and iterating through multiple perspectives to create a thorough impact assessment model.

 While the article provides a comprehensive overview of the methodology and its steps, some details could be expanded to facilitate replication. For instance, more specific examples of how expert feedback was integrated and the exact nature of the iterative process could be clarified.

 Points to Improve:

 1. While the methodology is well-described, providing additional specifics on the iterative process would enhance replicability. For example, detailed descriptions of the expert feedback sessions, including how feedback was solicited, processed, and integrated into the impact diagrams, would be useful. Including templates or tools used in the literature review and interrelation assessment phases would help other researchers replicate the study more precisely.

 2. The article mentions the use of interrelation assessment to identify potential feedback loops and additional impact areas. A more detailed explanation of this process would strengthen this section.

 3. The process of extending and improving the initial impact area diagram through interrelation assessment and grouping is crucial. However, the article could benefit from more detailed examples of how specific impact areas were expanded upon or refined based on this method.

 While the study provides a solid foundation for assessing the impacts of CAVs, replication by other researchers is necessary to validate, refine, and potentially standardize the methodology. This process will help ensure that the proposed framework is robust, widely applicable, and beneficial for policymakers and stakeholders involved in the deployment and regulation of CAV technologies.

Is the rationale for developing the new method (or application) clearly explained?

Yes

Is the description of the method technically sound?

Yes

Are the conclusions about the method and its performance adequately supported by the findings presented in the article?

Yes

If any results are presented, are all the source data underlying the results available to ensure full reproducibility?

No source data required

Are sufficient details provided to allow replication of the method development and its use by others?

Partly

Reviewer Expertise:

Connected and Automated Vehicles; Impact Assessment Methodologies; Transport Systems and Mobility; Transport Policy

I confirm that I have read this submission and believe that I have an appropriate level of expertise to confirm that it is of an acceptable scientific standard, however I have significant reservations, as outlined above.

# Reviewer report: Reviewer response for version 3

**DOI:** 10.21956/openreseurope.17982.r35260

**Published:** 2023-10-17

**Authors:** Yvonne Barnard

**Affiliations:** 1 Institute for Transport Studies, University of Leeds, Leeds, UK

The article has been improved taking into account comments. It is an important contribution to impact assessment of automated mobility.

Is the rationale for developing the new method (or application) clearly explained?

Partly

Is the description of the method technically sound?

Partly

Are the conclusions about the method and its performance adequately supported by the findings presented in the article?

Partly

If any results are presented, are all the source data underlying the results available to ensure full reproducibility?

No source data required

Are sufficient details provided to allow replication of the method development and its use by others?

Yes

Reviewer Expertise:

connected and automated mobility, evaluation methodology for field operational tests, user acceptance, impact assessment

I confirm that I have read this submission and believe that I have an appropriate level of expertise to confirm that it is of an acceptable scientific standard.

# Reviewer report: Reviewer response for version 2

**DOI:** 10.21956/openreseurope.15890.r29023

**Published:** 2022-05-16

**Authors:** Yvonne Barnard

**Affiliations:** 1 Institute for Transport Studies, University of Leeds, Leeds, UK

The article has been improved by the changes the authors made. I still struggle with the concept “structured holistic approach”. The amended text “To obtain such a comprehensive overview of all impacts, a structured holistic approach is needed. E.g., structuring the impact assessment process with the goal of obtaining a holistic set of potential impacts” does not clarify this for me. Holistic is a vague term, and what does structuring the impact assessment process mean, normally impact assessment is structured, projects do usually not undertake a chaotic or random approach. It would help if a paragraph could be added explaining what is wrong with current approaches, and how this could be improved.

 In the literature review papers like Chan 2017 are criticised for not providing a structured holistic approach, although there is a categorization. From this, the reader may be able to conclude that the structured holistic approach requires something like a model, but it is rather implicit.

 In reaction to my question about the distinction between comfort and convenience the reaction of the authors (“The difference between comfort and convenience is that the former relates to a physical state of the vehicle user (e.g., sitting in a comfortable chair during a long distance travel) and the latter relates to ease of use (e.g., being at work quicker due to a shorter travel time”) does not really help to understand why commuting distances are put under comfort. Please give some explanation in the text.

 The authors have changed the text about the completeness of the diagram, which is an improvement. However, I still think that completeness and validation of completeness is an elusive concept, and something more could have been said on the need to regularly revisit and update the diagram.

 The article certainly is interesting and a good contribution to the difficult area of impact assessment. The article could be fully approved when more clarification and definitions are given of the main concept of "structured holistic approach".

Is the rationale for developing the new method (or application) clearly explained?

Partly

Is the description of the method technically sound?

Partly

Are the conclusions about the method and its performance adequately supported by the findings presented in the article?

Partly

If any results are presented, are all the source data underlying the results available to ensure full reproducibility?

No source data required

Are sufficient details provided to allow replication of the method development and its use by others?

Yes

Reviewer Expertise:

connected and automated mobility, evaluation methodology for field operational tests, user acceptance, impact assessment

I confirm that I have read this submission and believe that I have an appropriate level of expertise to confirm that it is of an acceptable scientific standard, however I have significant reservations, as outlined above.

# Response

**Published:** 2023-09-18

**Authors:** Diane Cleij

**Affiliations:** SWOV, The Netherlands

Dear reviewer, Thank you for reviewing the article and providing valuable feedback. We have addressed the concerns you expressed to the best of our abilities and are of the opinion that it significantly improved the article. Especially your concerns regarding the difference of the presented approach with respect to other approaches from literature brought new insights for us, which are represented by the significant changes made to the article. In the text below your comments are shown in bold font, our response is shown below in plain text and if any adjustments were made to the text this is shown in italic, with the exact adjustments underlined.  We hope the revisions address your original reservations sufficiently. We hope you can find the time to review the article once more. Kind regards, on behalf of all authors, Diane Cleij  

**Reviewer comment 1** ***The article has been improved by the changes the authors made. I still struggle with the concept “structured holistic approach”. The amended text “To obtain such a comprehensive overview of all impacts, a structured holistic approach is needed. E.g., structuring the impact assessment process with the goal of obtaining a holistic set of potential impacts” does not clarify this for me. Holistic is a vague term, and what does structuring the impact assessment process mean, normally impact assessment is structured, projects do usually not undertake a chaotic or random approach. It would help if a paragraph could be added explaining what is wrong with current approaches, and how this could be improved.*** Thank you for this constructive feedback. We have reviewed our approach and came to the conclusion that the main contribution is the combination of multiple methods that each view the identification of impact areas from a different perspective, iterating though these methods to minimize the omission of impact areas and keeping a broad initial scope when starting the impact area assessment. The different methods we employed are: literature search, project team feedback, interrelation assessment and grouping. We added this perspective throughout the paper, which resulted in major textual changes in the introduction.   We added the following explanation for a holistic approach: *“To obtain a comprehensive overview of all impact areas, a holistic approach for defining impact areas is needed. E.g., an impact area assessment approach that, rather than focussing on specific impact areas in isolation, focusses on the whole set of impact areas and their interrelations with the goal of obtaining a complete overview of impact areas.* *“* We added more information about the specifics of our approach at the end of the introduction: “ *This paper presents the approach taken in LEVITATE to explore and define impact areas and their interrelations as a starting point for quantitative impact assessment. The modelling approach shows many similarities with the modelling of causal loop diagrams ( Bala et al., 2017). The approach includes iterations between four distinct methods: literature research,* *project team feedback* *, interrelation assessment and grouping. Each of these methods provides a different perspective on potential missing impact areas. In addition, the scope is initially kept relatively broad as to not constrain the identification of impact areas and increase the chances of identifying relevant interrelations. The combination of an iterative approach, combining multiple perspectives and initially retaining a broad scope is expected to provide a more holistic overview of impact areas than when adopting only one of these methods and/or limiting the scope beforehand.”* We added information on this view of the approach to the discussion: *“ The approach presented here combines four distinct methods: literature research, project team feedback, interrelation assessment and grouping. The scope is initially kept relatively broad as to not constrain the identification of impact areas and increase the chances of identifying relevant interrelations.* *The main difference between the approach presented here and those presented in ( Innamaa et al., 2018b) and ( Milakis et al., 2017) is the combination of looking at the impact areas from different perspectives by adopting multiple methods and iterating through these methods to minimize the omission of impact areas and with that obtain a holistic overview of impact areas. “*

**Reviewer comment 2** ***In the literature review papers like Chan 2017 are criticised for not providing a structured holistic approach, although there is a categorization. From this, the reader may be able to conclude that the structured holistic approach requires something like a model, but it is rather implicit. *** We agree that our critics on other literature was not substantiated enough in the article. We now added for each discussed article a more elaborate explanation of what is missing in the approaches used in those articles in our opinion. This resulted in the following additions and changes to the introduction: *“Most overviews of impact areas in previous literature consist of written summaries based on literature research. They often provide a categorization of the impact areas generally defined by the authors themselves. In ( Fagnant & Kockelman, 2015) impacts are first discussed under four headings: safety, congestion and traffic operations, travel behaviour impacts and freight transport. Subsequently, they present estimates of societal and personal economic benefits based on literature findings of expected changes in vehicle miles travelled, vehicle ownership, technology cost, crash rates, congestion reduction and parking. In ( Hörl et al., 2016) the impacts of vehicle automation are categorized as impacts on mobility, city planning, car industry, work organisation, user profiles, delivery of goods and price. Within each category many more specific impacts and some interrelations are mentioned. In ( Chan, 2017) benefits, i.e., positive impacts, of automated vehicles are categorized under vehicle user, transportation operation and society perspectives. Many more overviews, generally based on literature research, can be found ( Herrmann et al., 2018; Kockelman & Boyles, 2018; Polis, 2018). Most of these articles and reports do not provide much insight into exactly how these overviews were obtained, other than mentioning literature research was performed.* *A more structured and holistic approach was taken in ( Milakis et al., 2017 ). The authors first developed a simplified concept which represented the possible implications of automated vehicles and identified impact areas and their respective mechanisms based on their own analytical thinking. They then performed a structured literature review on the impacts of automated vehicles . By comparing their own concept and identified impacts to those found in literature, they then identify research gaps. Their concept consists of four concentric circles showing vehicle automation technology in the centre. The first order impacts of this vehicle technology on the transport system that are directly noticed by the road users are shown around this centre, followed by the second order impacts on, for example, infrastructure and land use in the third circle band. Finally, in the fourth circle band, the wider societal impacts are shown. The model attempts to show the propagation of vehicle technology impacts from direct impacts on road users to societal impacts, giving a more coherent view of the relationship between impacts.* *In contrast to the previously mentioned studies based on literature review only, the study described by Milakis et al (2017) approaches the challenges of creating a holistic overview of impact areas from two distinct perspectives, i.e., the perspective based on expert knowledge in combination with analytical thinking from the authors and the perspective based on literature review. By combining these two perspectives, gaps in literature were found, implying that this method is more holistic than solely using literature review. However,* *the simplicity of the concept doesn’t allow for causal relations between specific impact areas to be investigated. It is possible that relevant secondary effects caused by these interrelations are therefore omitted. Additionally, as they did not report to have iterated between the two perspectives, the holism of the results strongly depends on the initial knowledge level of the experts.* *A more elaborate approach is taken in ( Innamaa et al., 2018a; Innamaa et al., 2018b). They define nine impact groups that are displayed on a graph of spatial resolution vs. time frame. The direct impacts, those that have a relatively clear cause-effect relationship with the primary activity or action, are those of small spatial resolution and short time frame. These impacts can usually be measured in a field test and are grouped under safety, vehicle operations, personal mobility and energy/emissions. Indirect impacts, on the other hand, are defined as resulting from these direct impacts and can often not be measured in a field test. They include impacts on network efficiency, travel behaviour, public health, infrastructure and land use and socio-economic impacts.* *In a first step of the impact analysis approach described by Innamaa et al. (2018a, 2018b) they perform a classification of the system and the design domain. In this step they, for example, make clear which automated functions and services will be included in the impact analysis. For the impact evaluation they then propose charts indicating potential impact paths starting from direct impacts on vehicle operations, driver or traveller, quality of travel and transport system and leading to one of the previously mentioned impact areas, such as safety. In addition, they recommend not only investigating these one-way paths to the impact areas, but also the strong links between the impact areas. As a next step, they recommend elaborating further on the proposed impact paths for the system under evaluation by adding direction of change, similar to what is done in causal-loop-diagrams.* *In contrast to ( Milakis et al., 2017), the approach described in Innamaa et al. (2018a, 2018b) does have a strong focus on interrelations between impact areas. The authors do recommend to further assess indirect impacts. Rather than the elaborate literature review presented in (* *Milakis et al., 2017* *), however, this approach starts from the impact areas defined in one overview study (Smith et al. 2015). The impact areas and interrelations were later refined at several conferences and meetings. The articles do not elaborate on how either the further assessment of indirect impacts or the mentioned refinements should be or were be performed.”*

**Reviewer comment 3** ***In reaction to my question about the distinction between comfort and convenience the reaction of the authors (“The difference between comfort and convenience is that the former relates to a physical state of the vehicle user (e.g., sitting in a comfortable chair during a long distance travel) and the latter relates to ease of use (e.g., being at work quicker due to a shorter travel time”) does not really help to understand why commuting distances are put under comfort. Please give some explanation in the text.*** We have reconsidered out previous response and agree with the reviewer. The commuting distance could be placed both under comfort and convenience. We realized that the reason for not duplicating any impact areas in the grouping step was not explained. We added such explanation to the beginning of the section on “Impact diagram elaboration”: “ *The resulting impact area diagrams can become quite complex, containing a large amount of identified impact areas and interrelations. A second, step is therefore performed to create a clearer overview of the impact areas and identify which group of impact areas has potentially not been sufficiently explored. In this step the impact areas are grouped along dimensions commonly found in the literature.* “ We addressed the lack of duplication of the commuting distance in the grouping diagram specifically in the end of this section when Figure 2 is introduced. *“ It is possible that impact areas can logically be placed in multiple groups. Commuting distances, for example, can affect both convenience and comfort. One wat to address this is to duplicate this impact area and place it in both groups. Alternatively, the impact area can be placed in the group that contains the least impact areas. Here we chose the latter, as the closely related impact area of travel time was already present in the group “Convenience” and duplication would increase the complexity of the overview. To extend the impact diagram, each of these subgroups was analyzed for missing impact areas and newly found impacts were added to the overall impact area diagram. Figure 2 , for example, implies that the groups “Cost” and “Comfort” are potentially not sufficiently explored and require further attention. Methods of literature research or project team feedback* *with a focus on impacts related to these groups can be adopted to identify additional impact areas within these groups* *.*”

**Reviewer comment 4** ***The authors have changed the text about the completeness of the diagram, which is an improvement. However, I still think that completeness and validation of completeness is an elusive concept, and something more could have been said on the need to regularly revisit and update the diagram.*** We agree with the reviewer that this advice would be a good addition and added it to the discussion section: “ *While the method aims to be as holistic as possible in defining the impact areas, it is not possible to know if true completeness is achieved . It is therefore advised to continue to regularly revisit and update the diagram if necessary.* “

**Reviewer comment 5** ***The article certainly is interesting and a good contribution to the difficult area of impact assessment. The article could be fully approved when more clarification and definitions are given of the main concept of "structured holistic approach".*** Thank you for seeing the value in our contribution. We hope the changes we made are sufficient.

# Reviewer report: Reviewer response for version 2

**DOI:** 10.21956/openreseurope.15890.r29024

**Published:** 2022-05-09

**Authors:** Satu Innamaa

**Affiliations:** 1 VTT Technical Research Centre of Finland Ltd., Espoo, Finland

Your revised article (version 2) entitled ”First steps towards a holistic impact assessment methodology for connected and automated vehicles” has improved since the original version. However, there are still some issues that should, in my opinion, be addressed. Therefore, my recommendation is to approve the article with reservation and the following comments should be taken into account before the final approval.

- As a generic remark, you talk throughout the paper of ‘impacts’ when you refer to ‘impact areas’. In my opinion, e.g. ‘Congestion’ or ‘Public health’ are names of ***impact areas***, not impacts. Consequently, I would use the term ‘impact’ in this paper when really talking of an impact (an increase or a decrease in something), and use the term ‘impact area’ when talking only of the topics affected by automation. E.g. in the abstract, you write “ *In this paper a methodology is presented to explore and define these impacts…*” but ‘Impact areas’ would be a more precise term. Check the use of the term ‘impact’ throughout the paper to ensure that the reader has correct expectations on the content of the paper.
- In the introduction (1 ^st^ paragraph), you write “ *Most vehicles on the roads today can take over part of the driving task…*”. In fact, the penetration rates of different ADAS are still rather small when looking at the whole European car fleet today even ADAS is getting more common among the new cars. Therefore, I would use e.g. ‘More and more vehicles’ or ‘Many newly registered vehicles’ instead of ‘Most vehicles’. A good reference should also be added for the statement.
- You say in the 3 ^rd^ paragraph of the introduction that “ *structured holistic approaches to impact assessment of automated vehicles are scarce*”. For this statement, it would be good to provide the characteristics that you require for the impact assessment approach to classify it as ‘an structured holistic approach’. Is a definition of the connections between impact areas (causal loop diagram) required? In my opinion, impact assessment can be structured and holistic also without CLD. You have the same statement also in the review chapter. Therefore, a definition of the requirements behind these statements is needed.
- Add the reference to the last paragraph of the Review chapter. Now ‘they’ in the paragraph refers to the authors referred to in the previous paragraph. This might not be obvious to the reader.
- In 1 ^st^ paragraph of the chapter ‘Impact assessment method’, there is ‘in’/’of’ missing from ‘reduction in/of travel time’.
- You write that you defined “ *a holistic set of starting points*” and that they were “ *vehicle design, level of automation and connectivity*”. Is this set “holistic”? How do you define it? Please, specify it in the paper.
- In Figure 2, you do not connect any of the impact areas with ‘Vehicle design’ or ‘Connectivity’. Shouldn’t at least ‘Driving behaviour’ be affected by ‘Connectivity’? Maybe also ‘Use and valuation of travel time’ with ‘Vehicle design’.
- In Table 2, you have classified ‘Land use’ under ‘Transport operations’. I see the impact area ‘Land use’ as much broader than that. Therefore, I would have placed it under ‘Society’.
- Subchapter ‘Ethics statement’ is now placed under ‘Impact assessment method’. In its current form, this statement could be moved to ‘Acknowledgements’.
- In the chapter ‘Method output…’, you have the sentence “ *These categories all refer to…*”. I think you mean here ‘Direct impacts’, and not all ‘direct, systematic and wider’. If so, please correct.
- In the 3 ^rd^ paragraph of the chapter ‘Further steps…’, you state “ *The impact diagram can be used to define relevant use cases…*”. Do you mean *use cases of automated driving*? How do you define them based on figures 6 and 7? If you meant something else, please, specify it.
- Later you also say that “ *Figure 6 shows… they will probably have a lower risk of being involved in a crash…*”. How do you see in Figure 6 that the crash risk is lower? Impact area ‘safety’ does not automatically mean ‘lower crash risk’, even if this is an impact that we hope to see with driving automation. Thus, reconsider the sentence structure and separate what you see from the figure from assumptions not seen from the figure. You can modify it e.g. to “If we assume that the crash risk is lower…”.
- In Figure 5 ‘secondary impacts’ are indicated to be related to ‘behavioural adaptation’. You have many items related to ‘costs’ here. I understand the cost factors to be part of impact generators. However, direct impacts on costs I would not say to be part of behavioural adaptation. Consequently, I would remove the brackets from the figure heading and call it simply ‘secondary impacts’. The same suggestion is also for Figure 7.

Is the rationale for developing the new method (or application) clearly explained?

Yes

Is the description of the method technically sound?

Yes

Are the conclusions about the method and its performance adequately supported by the findings presented in the article?

Partly

If any results are presented, are all the source data underlying the results available to ensure full reproducibility?

Yes

Are sufficient details provided to allow replication of the method development and its use by others?

Yes

Reviewer Expertise:

Impact assessment, connected and automated driving, transport, mobility

I confirm that I have read this submission and believe that I have an appropriate level of expertise to confirm that it is of an acceptable scientific standard, however I have significant reservations, as outlined above.

# Response

**Published:** 2023-09-18

**Authors:** Diane Cleij

**Affiliations:** SWOV, The Netherlands

Dear reviewer, Thank you for reviewing the article and providing valuable feedback. We have addressed the concerns you expressed to the best of our abilities and are of the opinion that it significantly improved the article. In the text below your comments are shown in bold font, our response is shown below in plain text and if any adjustments were made to the text this is shown in italic, with the exact adjustments underlined.  We hope the revisions address your original reservations sufficiently. Kind regards, on behalf of all authors, Diane Cleij

**Reviewer comment 1** **“Your revised article (version 2) entitled ”First steps towards a holistic impact assessment methodology for connected and automated vehicles” has improved since the original version. However, there are still some issues that should, in my opinion, be addressed. Therefore, my recommendation is to approve the article with reservation and the following comments should be taken into account before the final approval.** **As a generic remark, you talk throughout the paper of ‘impacts’ when you refer to ‘impact areas’. In my opinion, e.g. ‘Congestion’ or ‘Public health’ are names of impact areas, not impacts. Consequently, I would use the term ‘impact’ in this paper when really talking of an impact (an increase or a decrease in something), and use the term ‘impact area’ when talking only of the topics affected by automation. E.g. in the abstract, you write “In this paper a methodology is presented to explore and define these impacts…” but ‘Impact areas’ would be a more precise term. Check the use of the term ‘impact’ throughout the paper to ensure that the reader has correct expectations on the content of the paper.”** Thank you for your comment. We agree with the reviewer and made the proposed adjustments to the terminology in the paper.

**Reviewer comment 2** **“In the introduction (1st paragraph), you write “Most vehicles on the roads today can take over part of the driving task…”. In fact, the penetration rates of different ADAS are still rather small when looking at the whole European car fleet today even ADAS is getting more common among the new cars. Therefore, I would use e.g. ‘More and more vehicles’ or ‘Many newly registered vehicles’ instead of ‘Most vehicles’. A good reference should also be added for the statement.”** We agree with the reviewer and made the suggested adjustment.

**Reviewer comment 3** **“You say in the 3rd paragraph of the introduction that “structured holistic approaches to impact assessment of automated vehicles are scarce”. For this statement, it would be good to provide the characteristics that you require for the impact assessment approach to classify it as ‘an structured holistic approach’. Is a definition of the connections between impact areas (causal loop diagram) required? In my opinion, impact assessment can be structured and holistic also without CLD. You have the same statement also in the review chapter. Therefore, a definition of the requirements behind these statements is needed.”** Thank you for this constructive feedback, we agree with the reviewer that this needs more clarification. We have reviewed our approach and came to the conclusion that the main contribution of our approach in making it more holistic is the combination of multiple methods that each view the identification of impact areas from a different perspective, iterating though these methods to minimize the omission of impact areas and keeping a broad initial scope when starting the impact area assessment. The different methods we employed are: literature search, project team feedback, interrelation assessment and grouping. We added this perspective throughout the paper, which resulted in major textual changes in the introduction.  We added the following explanation for a holistic approach: *“To obtain a comprehensive overview of all impact areas, a holistic approach for defining impact areas is needed. E.g., an impact area assessment approach that, rather than focussing on specific impact areas in isolation, focusses on the whole set of impact areas and their interrelations with the goal of obtaining a complete overview of impact areas.* *“* We added more information about the specifics of our approach at the end of the introduction: “ *This paper presents the approach taken in LEVITATE to explore and define impact areas and their interrelations as a starting point for quantitative impact assessment. The modelling approach shows many similarities with the modelling of causal loop diagrams (* *Bala et al., 2017* *). The approach includes iterations between four distinct methods: literature research, project team feedback* *, interrelation assessment and grouping. Each of these methods provides a different perspective on potential missing impact areas. In addition, the scope is initially kept relatively broad as to not constrain the identification of impact areas and increase the chances of identifying relevant interrelations. The combination of an iterative approach, combining multiple perspectives and initially retaining a broad scope is expected to provide a more holistic overview of impact areas than when adopting only one of these methods and/or limiting the scope beforehand*” We added information on this view of the approach to the discussion: *“ The approach presented here combines four distinct methods: literature research, project team feedback, interrelation assessment and grouping. The scope is initially kept relatively broad as to not constrain the identification of impact areas and increase the chances of identifying relevant interrelations.* *The main difference between the approach presented here and those presented in ( Innamaa et al., 2018b) and ( Milakis et al., 2017) is the combination of looking at the impact areas from different perspectives by adopting multiple methods and iterating through these methods to minimize the omission of impact areas and with that obtain a holistic overview of impact areas. “*

**Reviewer comment 4** ***“Add the reference to the last paragraph of the Review chapter. Now ‘they’ in the paragraph refers to the authors referred to in the previous paragraph. This might not be obvious to the reader.”*** Thank you for this suggestion. We adjusted this in the text as follows: “ *In a first step of the impact analysis approach described by Innamaa et al. (2018a, 2018b) they perform a classification of the system and the design domain.“*

**Reviewer comment 5** ***“In 1st paragraph of the chapter ‘Impact assessment method’, there is ‘in’/’of’ missing from ‘reduction in/of travel time’.”*** Thank you, we corrected it.

**Reviewer comment 6** ***“You write that you defined “a holistic set of starting points” and that they were “vehicle design, level of automation and connectivity”. Is this set “holistic”? How do you define it? Please, specify it in the paper.”*** Thank you for your comment. As mentioned in the reply to reviewer comment 1, we reviewed our approach. While we aimed to be as complete as possible when setting up the impact diagram and its impact generators, we cannot claim at that stage that it was complete (or as we wrote “holistic”). The holism of the approach is achieved through the iterations between perspectives as elaborated on in the reply to reviewer comment 1 and the corresponding adjustments to the article. We therefore adjusted to text referred to in reviewer comment 6 as follows: “ *To structure the diagram and define an initial set of starting points generating these impacts, the top of the diagram contains the technological changes that drive the impacts; the impact generators*.”

**Reviewer comment 7** ***“In Figure 2, you do not connect any of the impact areas with ‘Vehicle design’ or ‘Connectivity’. Shouldn’t at least ‘Driving behaviour’ be affected by ‘Connectivity’? Maybe also ‘Use and valuation of travel time’ with ‘Vehicle design’.”*** Thank you for your comment, it made us realize that we did not explain this figure adequately. This figure shows a simplified example of a first set of up of the impact area diagram that clearly misses important impact areas related to, for example, vehicle design and connectivity. We adjusted the text on two points. In the impact area set up section when introducing the figure we adjusted the text as follows: “ *A simplified example of such an initial impact area diagram set up including only six impact areas is shown in Figure 2.* “ In the section on impact area diagram elaboration we adjusted the text in the first paragraph as follows: “ *During the interrelation assessment , each impact area in the diagram was analyzed for possible further relations to other impact areas in the diagram and impact areas not yet in the diagram. In* *Figure 2* *, for example, no interrelations between two of the impact generators was found and this could therefore be a first clue that impact areas related to these impact generators are missing. In search for such additional impact areas, additional literature was often consulted.* “

**Reviewer comment 8** ***“In Table 2, you have classified ‘Land use’ under ‘Transport operations’. I see the impact area ‘Land use’ as much broader than that. Therefore, I would have placed it under ‘Society’.”*** We agree with the reviewer and placed “Land use” within the group “Society”.  

**Reviewer comment 9** ***“Subchapter ‘Ethics statement’ is now placed under ‘Impact assessment method’. In its current form, this statement could be moved to ‘Acknowledgements’.”*** We agree with the reviewer that the ethics statement feels a bit misplaced and moved it to the end of the article. 

**Reviewer comment 10** ***“In the chapter ‘Method output…’, you have the sentence “These categories all refer to…”. I think you mean here ‘Direct impacts’, and not all ‘direct, systematic and wider’. If so, please correct.”*** We do refer to all three categories, as they all originate from the impact generators in the first layer of the diagram.

**Reviewer comment 11** ***“In the 3rd paragraph of the chapter ‘Further steps…’, you state “The impact diagram can be used to define relevant use cases…”. Do you mean use cases of automated driving? How do you define them based on figures 6 and 7? If you meant something else, please, specify it.”*** Thank you for that comment, we now see that this sentence is missing quite some context. The use cases that this sentence referred to were used in the backcasting work we did within LEVITATE. We replaced this sentence with the following text and moved it to the end of the section: *“* *Within the LEVITATE project, the focus was not only on forecasting impacts of automated vehicles, but also on backcasting, i.e., identifying policy measures that would result in desired impacts within a set time span. Within LEVITATE the impact area diagram was also used to brainstorm about relevant policy measures by providing details on interrelations between the desired areas of wider societal impact and the areas of the lower level direct impacts. For example, if a desired societal impact is reducing air pollution, the interrelations in* *Figure 4* *imply that one can, amongst others, develop policy interventions to influence the “vehicle design” (which in turn influences the “energy efficiency”) or to influence “connectivity” (which in turn has an effect on “road capacity” and subsequently on “congestion”). Based on such analyses a set of relevant policy interventions for further detailed analysis can be defined.* “

**Reviewer comment 12** ***“Later you also say that “Figure 6 shows… they will probably have a lower risk of being involved in a crash…”. How do you see in Figure 6 that the crash risk is lower? Impact area ‘safety’ does not automatically mean ‘lower crash risk’, even if this is an impact that we hope to see with driving automation. Thus, reconsider the sentence structure and separate what you see from the figure from assumptions not seen from the figure. You can modify it e.g. to “If we assume that the crash risk is lower…”.”*** We agree that it is not known if automated vehicles have a positive impact on road safety and that this also not visible from the diagram. We adjusted the text as follows: “ *Figure 6 shows that automated vehicles affect road safety directly (primary impact) via many routes, for example , as automated vehicles have different capabilities and limitations as compared to human driven vehicles, they will likely also have a different risk of being involved in a crash than human driven vehicles. The risk changes likely increase with increasing level of automation , as the human involvement decreases. This impact is indicated in Figure 6 with the arrow between “level of automation” and “road safety”. If vehicles are able to communicate with each other, i.e. if they are connected (CAVs), the risk of a crash will also be affected. This additional change to road safety is indicated with the arrow between “connectivity” and “road safety”.* *“*

**Reviewer comment 13** ***“In Figure 5 ‘secondary impacts’ are indicated to be related to ‘behavioural adaptation’. You have many items related to ‘costs’ here. I understand the cost factors to be part of impact generators. However, direct impacts on costs I would not say to be part of behavioural adaptation. Consequently, I would remove the brackets from the figure heading and call it simply ‘secondary impacts’. The same suggestion is also for Figure 7.”*** We agree and adjusted the title as recommended.

# Reviewer report: Reviewer response for version 1

**DOI:** 10.21956/openreseurope.14954.r27787

**Published:** 2021-11-15

**Authors:** Yvonne Barnard

**Affiliations:** 1 Institute for Transport Studies, University of Leeds, Leeds, UK

The paper addresses an important topic: how to study the possible impacts of road automation. The problem with studying the impacts is that although the technology is developing, the percentage of automated vehicles on the road is still very low: the first sentence in the abstract is somewhat exaggerated.  The use of the term connected and automated vehicles is that it seems to encompass everything from driver support systems to robot-taxis, and this does not help when looking at impacts. The major shift in societal impacts may come from widespread full or nearly full automation and not from driving with systems that perform some driving functions automatically under control of the driver. It is not always clear in the paper about what level of automation and what level of penetration the impacts are discussed.

 Some justification for why “ *as many impacts*” is the focus of the work would have been helpful. If we look at wider societal impacts there may be an infinite range of possible changes as it is not only automation in mobility that is changing, but society as a whole is changing as well, which will in turn impact mobility (economic developments, climate change consequences, energy, automation in other sectors, etc.). In order to provide policy support, as is the aim of the project, I’m not sure if “ *as many*” is going to be very helpful.

 It would have been good to have some more clarity about the terms used and the differences between a “ *structured*” approach, a “ *holistic*” approach, a “ *structured holistic approach*”, between causal loop diagrams and feedback loops.

 Sometimes the text is rather vague, for example, directly under the heading “Impact assessment method” it says “ *The focus is put on the system as a whole from the start, including feedback and interrelations between different impact areas*”. It is not clear to me what “ *the system as a whole*” is, is it traffic, mobility, society….? Also, it is not clear whether the focus is on vehicles themselves or on the wider mobility system. In table 1 both are being described.

 I am wondering about “ *The main groups in the taxonomy described in (Chan, 2017) was deemed most holistic as it encompassed all others*.” This taxonomy is the most high-level, simple one, but why call it holistic? What is the difference between comfort and convenience? In the example in figure 2 commuting distance is comfort and travel time convenience. I don’t understand why.

 I have doubts about the claim that the project aims to the completeness of the impact diagram, is this ever possible? The claim “ *Therefore, a validation of the completeness of the diagram was approximated by comparing the impact diagrams to impacts found in additional literature, in combination with a final review by project members. The literature used for this validation (Litman, 2019; Sousa et al., 2018; van Nes & Duivenvoorden, 2017) was not part of the initial explorative literature review. No additional impacts or interrelations were found and therefore the completeness of the diagram was deemed sufficiently validated*.” Can you really validate completeness by looking at a few extra literature sources?

 The section in the discussion is somewhat confusing: “ *This approach to impact assessment has its limitations. While the method aims to be as holistic as possible in defining the impacts, it is not possible to know if true completeness is achieved. Aiming for completeness helps to create insight in all the different factors that are interrelated and together define impacts of CAVs. To achieve this, however, the scope of the assessment is initially kept quite large. This large scope makes it harder to be specific on the exact parameters and dose response curves needed to define each impact. After the scope has been reduced, as is proposed as a next step, many more steps will need to be taken before a quantitative impact assessment can be performed. Defining a smaller scope initially can make the overall process faster, but increases the chances of failing to identify certain relevant impacts and interrelations.*” Is the model complete or not, as claimed earlier in the paper. Is now the scope too large and has it to be made smaller, which seems to go against the objective of the project. What is then the difference with the other approaches in the literature?

 The conclusion states that “ *this method has proven successful for the purposes of the European project LEVITATE and can be expected to help others with similar analysis challenges*.” But not much is said about why it is successful and how that was established.

 In summary, in my opinion, the paper needs to provide more clarification of the terms being used. More explanation and justification are needed about the choices being made. More explanation is needed about the limits of the approach and the model, and the way in which they are going to be used and evaluated.

Is the rationale for developing the new method (or application) clearly explained?

Partly

Is the description of the method technically sound?

Partly

Are the conclusions about the method and its performance adequately supported by the findings presented in the article?

Partly

If any results are presented, are all the source data underlying the results available to ensure full reproducibility?

No source data required

Are sufficient details provided to allow replication of the method development and its use by others?

Yes

Reviewer Expertise:

connected and automated mobility, evaluation methodology for field operational tests, user acceptance, impact assessment

I confirm that I have read this submission and believe that I have an appropriate level of expertise to confirm that it is of an acceptable scientific standard, however I have significant reservations, as outlined above.

# Response

**Published:** 2022-04-06

**Authors:** Diane Cleij

**Affiliations:** SWOV, The Netherlands

Dear Dr. Barnard,

 Thank you for your comments, we feel they have improved the article significantly. In the text below we addressed your comments and indicate corresponding changes to the article text. Your comments are shown in grey, our response is shown below in black and the corresponding adjustments to the article text are shown in red italic. We hope the revisions address your original reservations sufficiently.

 Kind regards,

 Diane Cleij

**Reviewer Comment 1:**

**“The paper addresses an important topic: how to study the possible impacts of road automation. The problem with studying the impacts is that although the technology is developing, the percentage of automated vehicles on the road is still very low: the first sentence in the abstract is somewhat exaggerated.  The use of the term connected and automated vehicles is that it seems to encompass everything from driver support systems to robot-taxis, and this does not help when looking at impacts. The major shift in societal impacts may come from widespread full or nearly full automation and not from driving with systems that perform some driving functions automatically under control of the driver. It is not always clear in the paper about what level of automation and what level of penetration the impacts are discussed.“**

 Thank you for your comment. It is true that the initial scoping is kept rather broad and can therefore be perceived as somewhat vague. As mentioned in the discussion, the scope was initially kept relatively broad, to avoid limiting the impact of brainstorming too early in the process. The LEVITATE projects considers the impacts of different penetration rates of first and second generation automated vehicles which are further specified in the LEVITATE project. In addition, impacts are estimated for a number of Sub Use Cases that can be found in Table 1. To make this clearer in the paper we added the following text:  

*“Within the policy support tool, impacts are estimated for different penetration rates of first and second generation automated vehicles as well as a number of additional policy measures and technologies. The tool will quantify the impacts presented in this paper accordingly.”*    

**Reviewer Comment 2:**

**"Some justification for why “as many impacts” is the focus of the work would have been helpful. If we look at wider societal impacts there may be an infinite range of possible changes as it is not only automation in mobility that is changing, but society as a whole is changing as well, which will in turn impact mobility (economic developments, climate change consequences, energy, automation in other sectors, etc.). In order to provide policy support, as is the aim of the project, I’m not sure if “as many” is going to be very helpful.”**

 Thank you for this comment. It is true that “many” is not better per se. However, the aim of the LEVITATE project is to estimate all (societal) impacts of developments related to Connected and Automated mobility. This is the core of the project. The policy support tool that is being developed within the LEVITATE project, serves a wide range of policy makers. To serve each of them, as many societal impacts impacts as possible are included in the tool. However, policy makers can select only those impacts that are of interest to them to get a more focussed output. We adjusted the text to emphasize the wide range of policy makers that are the focus group of the LEVITATE project.  

**“** *The policy support tool that will be developed during this project is intended to enable a wide range of policy makers to select policy interventions and assess the impacts of automated vehicles in the short, mid and long term future under different circumstances.* **”**  

 Additionally, including such a wide range of impacts can help uncovering unintended impacts and feedback loops that possibly stay hidden when limiting the scope of your impact assessment. An example of this was given in the discussion: “ *It has been assumed ( Aria et al., 2016; Arnaout & Arnaout, 2014; Papadoulis et al., 2019), for example, that smaller time headways increase road capacity and therefore decrease congestion and travel time. This assumption, however, does not take into account the well-established fact that decreased travel time creates a feedback loop that in turn increases vehicle km travelled and may increase.”* Congestion. In a worst-case scenario, travel time is unchanged, but there are more vehicles on the road creating more pollution.  

**Reviewer Comment 3:**

**“It would have been good to have some more clarity about the terms used and the differences between a “structured” approach, a “holistic” approach, a “structured holistic approach”, between causal loop diagrams and feedback loops.”**

 The authors agree that these terms benefit from some additional explanation. We adjusted the text as follows:  

*"To obtain such a comprehensive overview of all impacts, a structured holistic approach is needed. E.g., structuring the impact assessment process with the goal of obtaining a holistic set of potential impacts."* 

 Causal loop diagrams contain both feedforward and feedback loops. Where the former is the impact that happens first after the initial change in circumstances (e.g., introduction of CAV) and the latter loop is a consequence of this impact that feeds back to an earlier impact. For example, Introduction of CAVs could reduce travel time (feedforward loop) as they might make more efficient use of the road available due to shorter time headways. However, when travel time is lower, more people might use the car which will increase traffic flow and reduce traffic time again (feedback loop). To explain this more clearly in the article the following changes have been made:

*“The focus is put on the system as a whole from the start, thus including both feedforward and feedback casual relations between different impact areas. Examples of such causal relations are shown in Figure 1. Here the feedforward, or direct, relation is the potential impact of CAV regarding the reduction travel time due to the adoption of shorter time headways. The feedback relation is then the relation between increased traffic flow due to this shorter travel time that in turn increases the travel time.*

*Figure 1. Example of causal relations between impacts, containing both feedforward (green) and feedback relations (orange).*

*This on causal relations based approach to impact assessment can be used as an input in the development of specific use cases by focusing on the impacts that are of particular relevance in a specific use case.”*  

**Reviewer Comment 4:**

**“Sometimes the text is rather vague, for example, directly under the heading “Impact assessment method” it says “The focus is put on the system as a whole from the start, including feedback and interrelations between different impact areas”. It is not clear to me what “the system as a whole” is, is it traffic, mobility, society….? Also, it is not clear whether the focus is on vehicles themselves or on the wider mobility system. In table 1 both are being described.”**

 The system as a whole here refers to the inclusion of feedforward and feedback loops between impacts. To emphasize this we made the changes as shown in Reviewer comment 3. More generally speaking, the LEVITATE project looks at societal level impacts, but as they are fed by traffic and mobility level impacts these are included as well. To make this clearer we adjusted the text as follows:  

*“The LEVITATE project focuses on societal level impacts of CAVs in three areas of use: freight, urban and passenger car transport.”*  

**Reviewer Comment 5:**

**“I am wondering about “The main groups in the taxonomy described in (Chan, 2017) was deemed most holistic as it encompassed all others.” This taxonomy is the most high-level, simple one, but why call it holistic? What is the difference between comfort and convenience? In the example in figure 2 commuting distance is comfort and travel time convenience. I don’t understand why.”**

 It is a correct observation that the taxonomy described in (Chan, 2007) is the most high level one. This is likely why it is the most holistic, as it is surely easier to provide a holistic taxonomy defined on a higher level than on a lower level, where more details need to be reported to still be holistic. The difference between comfort and convenience is that the former relates to a physical state of the vehicle user (e.g., sitting in a comfortable chair during a long distance travel) and the latter relates to ease of use (e.g., being at work quicker due to a shorter travel time).  

**Reviewer Comment 6:**

**“I have doubts about the claim that the project aims to the completeness of the impact diagram, is this ever possible? The claim “Therefore, a validation of the completeness of the diagram was approximated by comparing the impact diagrams to impacts found in additional literature, in combination with a final review by project members. The literature used for this validation (Litman, 2019; Sousa et al., 2018; van Nes & Duivenvoorden, 2017) was not part of the initial explorative literature review. No additional impacts or interrelations were found and therefore the completeness of the diagram was deemed sufficiently validated.” Can you really validate completeness by looking at a few extra literature sources?”**

 The authors agree that an exact validation of completeness is not possible. For this reason the paper states that validation was approximated. The authors agree that more papers would of course improve such approximation. For the LEVITATE project, however, this level of validation was deemed sufficient. As this paper mainly aims to describe the method, it is up to those possibly using the method in the future to decide if for their project a more accurate approximation is needed.  

**Reviewer Comment 7:**

**The section in the discussion is somewhat confusing: “This approach to impact assessment has its limitations. While the method aims to be as holistic as possible in defining the impacts, it is not possible to know if true completeness is achieved. Aiming for completeness helps to create insight in all the different factors that are interrelated and together define impacts of CAVs. To achieve this, however, the scope of the assessment is initially kept quite large. This large scope makes it harder to be specific on the exact parameters and dose response curves needed to define each impact. After the scope has been reduced, as is proposed as a next step, many more steps will need to be taken before a quantitative impact assessment can be performed. Defining a smaller scope initially can make the overall process faster, but increases the chances of failing to identify certain relevant impacts and interrelations.” Is the model complete or not, as claimed earlier in the paper. Is now the scope too large and has it to be made smaller, which seems to go against the objective of the project. What is then the difference with the other approaches in the literature?”**

 As mentioned in the previous comment, the validation of completeness is an approximation. As is the case with all approximations, it can be wrong. For this model this would mean that we might miss some impacts that did not turn up in the validation phase, but would turn up if we would use more papers for the validation. However, for the purposes of this project the completeness was deemed sufficiently validated as stated in the article. As for the scope, this is only too large for the further steps in the process of quantifying the impacts, but not too large for the steps described in this article. Keeping the scope large in the initial stages of the process broadens researchers view on potential impacts, making it more likely that also less apparent potential causal relations are uncovered. As proposed in the article, one way of further scoping in later stages of the process is to select one primary impact area of interest. Then use the full scope impact diagram, which now also includes less apparent causal relations and impact areas, to select those impact areas that are of influence on this primary impact (e.g., in Figure 5 and 6 the impact area “Safety” was chosen). To make this clearer in the article we adjusted the text as follows: *“For example, one can decide to only look at safety impacts, while taking into account feedback loops caused by other types of impact that became apparent through the original broad scope diagram. “*  

**Reviewer Comment 8:**

**“The conclusion states that “this method has proven successful for the purposes of the European project LEVITATE and can be expected to help others with similar analysis challenges.” But not much is said about why it is successful and how that was established.”**

 We added the following text to the discussion to better explain the benefits of the method described in the article for the LEVITATE project:

*“Generally, the method presented here has helped structure the impact assessment process within the LEVITATE project, greatly benefitting the efficiency of our work. Furthermore, the relatively large scope in the first phase of impact assessment has benefitted the open exploration of potential impact areas and their interrelations. This strategy of delaying the definition of the final scope resembles the double diamond method often used in design processes (Tschimmel, 2012, Design Council 2015). Here a phase of exploration precedes the scoping phase so that first new insides are gathered and the problem is looked at in a fresh way before the final scoping occurs.* *”*

**Reviewer Comment 9:**

**“In summary, in my opinion, the paper needs to provide more clarification of the terms being used. More explanation and justification are needed about the choices being made. More explanation is needed about the limits of the approach and the model, and the way in which they are going to be used and evaluated.”**

 Thank you for your review, the authors hope that the previously described changes to the article address your concerns sufficiently.

# Response

**Published:** 2022-04-12

**Authors:** Diane Cleij

**Affiliations:** SWOV, The Netherlands

Dear Dr. Barnard, Thank you for your comments, we feel they have improved the article significantly. In the text below we addressed your comments and indicated corresponding changes to the article text. Your comments are shown in bold font, our response is shown below in plain text and if any adjustments were made to the text this is shown in italic, with the exact adjustments underlined. We hope the revisions address your original reservations sufficiently. Kind regards, Diane Cleij **Reviewer Comment 1:** **“The paper addresses an important topic: how to study the possible impacts of road automation. The problem with studying the impacts is that although the technology is developing, the percentage of automated vehicles on the road is still very low: the first sentence in the abstract is somewhat exaggerated.  The use of the term connected and automated vehicles is that it seems to encompass everything from driver support systems to robot-taxis, and this does not help when looking at impacts. The major shift in societal impacts may come from widespread full or nearly full automation and not from driving with systems that perform some driving functions automatically under control of the driver. It is not always clear in the paper about what level of automation and what level of penetration the impacts are discussed.“** Thank you for your comment. It is true that the initial scoping is kept rather broad and can therefore be perceived as somewhat vague. As mentioned in the discussion, the scope was initially kept relatively broad, to avoid limiting the impact brainstorming too early in the process. The LEVITATE projects considers impacts for different penetration rates of first and second generation automated vehicles which are further specified in the LEVITATE project. In addition, impacts are estimated for a number of Sub Use Cases that can be found in Table 1. To make this clearer in the paper we added the following text: *“ Within the policy support tool, impacts are estimated for different penetration rates of first and second generation automated vehicles as well as a number of additional policy measures and technologies. The tool will quantify the impacts presented in this paper accordingly.”* **Reviewer Comment 2:** **“Some justification for why “as many impacts” is the focus of the work would have been helpful. If we look at wider societal impacts there may be an infinite range of possible changes as it is not only automation in mobility that is changing, but society as a whole is changing as well, which will in turn impact mobility (economic developments, climate change consequences, energy, automation in other sectors, etc.). In order to provide policy support, as is the aim of the project, I’m not sure if “as many” is going to be very helpful.”** Thank you for this comment. It is true that “many” is not better per se. However, the aim of the LEVITATE project is to estimate all (societal) impacts of developments related to Connected and Automated mobility. This is the core of the project. The policy support tool that is being developed within the LEVITATE project, serves a wide range of policy makers. To serve each of them, as many societal impacts impacts as possible are included in the tool. However, policy makers can select only those impacts that are of interest to them to get a more focussed output. We adjusted the text to emphasize the wide range of policy makers that are the focus group of the LEVITATE project. “ *The policy support tool that will be developed during this project is intended to enable a wide range of policy makers to select policy interventions and assess the impacts of automated vehicles in the short, mid and long term future under different circumstances.* ” Additionally, including such a wide range of impacts can help uncovering unintended impacts and feedback loops that possibly stay hidden when limiting the scope of your impact assessment. An example of this was given in the discussion: “ *It has been assumed ( Aria et al., 2016; Arnaout & Arnaout, 2014; Papadoulis et al., 2019), for example, that smaller time headways increase road capacity and therefore decrease congestion and travel time. This assumption, however, does not take into account the well-established fact that decreased travel time creates a feedback loop that in turn increases vehicle km travelled and may increase congestion. In a worst-case scenario, travel time is unchanged, but there are more vehicles on the road creating more pollution. “* **Reviewer Comment 3:** **“It would have been good to have some more clarity about the terms used and the differences between a “structured” approach, a “holistic” approach, a “structured holistic approach”, between causal loop diagrams and feedback loops.”** The authors agree that these terms benefit from some additional explanation. We adjusted the text as follows: *“To obtain such a comprehensive overview of all impacts, a structured holistic approach is needed. E .g., structuring the impact assessment process with the goal of obtaining a holistic set of potential impacts. “* Causal loop diagrams contain both feedforward and feedback loops. Where the former is the impact that happens first after the initial change in circumstances (e.g., introduction of CAV) and the latter loop is a consequence of this impact that feeds back to an earlier impact. For example, Introduction of CAVs could reduce travel time (feedforward loop) as they might make more efficient use of the road available due to shorter time headways. However, when travel time is lower, more people might use the car which will increase traffic flow and reduce traffic time again (feedback loop). To explain this more clearly in the article the following changes have been made: *“The focus is put on the system as a whole from the start, thus including both feedforward and feedback casual relations between different impact areas . Examples of such causal relations are shown in Figure 1. Here the feedforward, or direct, relation is the potential impact of CAV regarding the reduction travel time due to the adoption of shorter time headways. The feedback relation is then the relation between increased traffic flow due to this shorter travel time that in turn increases the travel time.* *(for the added Figure see article)* *Figure 1. Example of causal relations between impacts, containing both feedforward (green) and feedback relations (orange).* *This on causal relations based approach to impact assessment can be used as an input in the development of specific use cases by focusing on the impacts that are of particular relevance in a specific use case.”* **Reviewer Comment 4:** **“Sometimes the text is rather vague, for example, directly under the heading “Impact assessment method” it says “The focus is put on the system as a whole from the start, including feedback and interrelations between different impact areas”. It is not clear to me what “the system as a whole” is, is it traffic, mobility, society….? Also, it is not clear whether the focus is on vehicles themselves or on the wider mobility system. In table 1 both are being described.”** The system as a whole here refers to the inclusion of feedforward and feedback loops between impacts. To emphasize this we made the changes as shown in Reviewer comment 3. More generally speaking, the LEVITATE project looks at societal level impacts, but as they are fed by traffic and mobility level impacts these are included as well. To make this clearer we adjusted the text as follows: *“The LEVITATE project focuses on societal level impacts of CAVs in three areas of use: freight, urban and passenger car transport.”* **Reviewer Comment 5:** **“I am wondering about “The main groups in the taxonomy described in (Chan, 2017) was deemed most holistic as it encompassed all others.” This taxonomy is the most high-level, simple one, but why call it holistic? What is the difference between comfort and convenience? In the example in figure 2 commuting distance is comfort and travel time convenience. I don’t understand why.”** It is a correct observation that the taxonomy described in (Chan, 2007) is the most high level one. This is likely why it is the most holistic, as it is surely easier to provide a holistic taxonomy defined on a higher level than on a lower level, where more details need to be reported to still be holistic. The difference between comfort and convenience is that the former relates to a physical state of the vehicle user (e.g., sitting in a comfortable chair during a long distance travel) and the latter relates to ease of use (e.g., being at work quicker due to a shorter travel time). **Reviewer Comment 6:** **“I have doubts about the claim that the project aims to the completeness of the impact diagram, is this ever possible? The claim “Therefore, a validation of the completeness of the diagram was approximated by comparing the impact diagrams to impacts found in additional literature, in combination with a final review by project members. The literature used for this validation (Litman, 2019; Sousa et al., 2018; van Nes & Duivenvoorden, 2017) was not part of the initial explorative literature review. No additional impacts or interrelations were found and therefore the completeness of the diagram was deemed sufficiently validated.” Can you really validate completeness by looking at a few extra literature sources?”** The authors agree that an exact validation of completeness is not possible. For this reason the paper states that validation was approximated. The authors agree that more papers would of course improve such approximation. For the LEVITATE project, however, this level of validation was deemed sufficient. As this paper mainly aims to describe the method, it is up to those possibly using the method in the future to decide if for their project a more accurate approximation is needed. **Reviewer Comment 7:** **“The section in the discussion is somewhat confusing: “This approach to impact assessment has its limitations. While the method aims to be as holistic as possible in defining the impacts, it is not possible to know if true completeness is achieved. Aiming for completeness helps to create insight in all the different factors that are interrelated and together define impacts of CAVs. To achieve this, however, the scope of the assessment is initially kept quite large. This large scope makes it harder to be specific on the exact parameters and dose response curves needed to define each impact. After the scope has been reduced, as is proposed as a next step, many more steps will need to be taken before a quantitative impact assessment can be performed. Defining a smaller scope initially can make the overall process faster, but increases the chances of failing to identify certain relevant impacts and interrelations.” Is the model complete or not, as claimed earlier in the paper. Is now the scope too large and has it to be made smaller, which seems to go against the objective of the project. What is then the difference with the other approaches in the literature?”** As mentioned in the previous comment, the validation of completeness is an approximation. As is the case with all approximations, it can be wrong. For this model this would mean that we might miss some impacts that did not turn up in the validation phase, but would turn up if we would use more papers for the validation. However, for the purposes of this project the completeness was deemed sufficiently validated as stated in the article. As for the scope, this is only too large for the further steps in the process of quantifying the impacts, but not too large for the steps described in this article. Keeping the scope large in the initial stages of the process broadens researchers view on potential impacts, making it more likely that also less apparent potential causal relations are uncovered. As proposed in the article, one way of further scoping in later stages of the process is to select one primary impact area of interest. Then use the full scope impact diagram, which now also includes less apparent causal relations and impact areas, to select those impact areas that are of influence on this primary impact (e.g., in Figure 5 and 6 the impact area “Safety” was chosen). To make this clearer in the article we adjusted the text as follows: *“For example, one can decide to only look at safety impacts, while taking into account feedback loops caused by other types of impact that became apparent through the original broad scope diagram. “* **Reviewer Comment 8:** **“The conclusion states that “this method has proven successful for the purposes of the European project LEVITATE and can be expected to help others with similar analysis challenges.” But not much is said about why it is successful and how that was established.”** We added the following text to the discussion to better explain the benefits of the method described in the article for the LEVITATE project: *“ Generally, the method presented here has helped structure the impact assessment process within the LEVITATE project, greatly benefitting the efficiency of our work. Furthermore, the relatively large scope in the first phase of impact assessment has benefitted the open exploration of potential impact areas and their interrelations. This strategy of delaying the definition of the final scope resembles the double diamond method often used in design processes (Tschimmel, 2012, Design Council 2015). Here a phase of exploration precedes the scoping phase so that first new insides are gathered and the problem is looked at in a fresh way before the final scoping occurs.* *”* **Reviewer Comment 9:** **“In summary, in my opinion, the paper needs to provide more clarification of the terms being used. More explanation and justification are needed about the choices being made. More explanation is needed about the limits of the approach and the model, and the way in which they are going to be used and evaluated.”** Thank you for your review, the authors hope that the previously described changes to the article address your concerns sufficiently.

# Reviewer report: Reviewer response for version 1

**DOI:** 10.21956/openreseurope.14954.r27698

**Published:** 2021-10-29

**Authors:** Satu Innamaa

**Affiliations:** 1 VTT Technical Research Centre of Finland Ltd., Espoo, Finland

The article ”Impact assessment methodology for connected and automated vehicles” by Cleij *et al.* presents a methodology to explore and define these impacts (specifically: impact areas) as starting point for quantitative impact assessment. The article presents the outcome of a systematic overview of the impact areas and linkages between them and some examples of how the result can be used.

 My recommendation is to approve the article with reservations taking into account the comments below:

- The article is named “Impact assessment methodology for connected and automated vehicles”. However, in your own words “This paper presents the approach … to explore and define impacts and their interrelations as a starting point for quantitative impact assessment”. Specifically, the paper focuses on “impact areas” rather than on “impacts” as an assessment of the magnitude or even the direction of the impact is not included. Thus, the title does not really correspond to the content as the full impact assessment methodology is not presented but only the potential impact areas affected by CAVs. Therefore, I would suggest to rename the article as something like “Holistic overview of impact areas for CAV”.
- In the abstract, you hint that “Results from the qualitative assessment … are presented”. However, if not even a direction of the impact is defined, can this be called “results of qualitative assessment”? I see this more as the identification of potential impact areas. Thus, I would recommend to clarify the content of the article for the abstract.
- Terms “non- *autonomous*, partial and fully *autonomous* vehicles” are used on page 4. The term “automated” would be better.
- On page 4 you state that “Beyond 25 years… mobility is expected to be “close to perfect”…”. Did you have a reference for this? Or is this a vision by LEVITATE? Such a strong statement would need clarification on this.
- The article uses the taxonomy suggested by Chan, grouping the impact areas under vehicle users (direct), transport operations (systemic impacts) and society (wider) which seems well justified and works well in the outcome.
- On page 7 you discuss “intended and unintended impacts” stating that all direct, systemic and wider impacts are “intended” while behavioral adaptation represents “unintended” impacts. I am not sure if this can be said. For example, the car manufacturer intends to make the car safe, comfortable, etc. but most likely does not “intend” to affect road capacity. On the other hand, they may intend to build the car such that it increases the trust to technology or provides accessibility to wider demographic groups than traditional cars. Now these items are listed under “unintended impacts”. I agree that it is important to cover both intended and unintended impacts but without knowing whether an impact is positive or negative it is hard to group the areas into these categories. In fact, under one impact area there may be “intended” and “unintended” impacts, for safety there may be an “intended” reduction in many types of accidents but also some “unintended” new types of accidents caused by automation. Thus, I would be cautious in categorizing the impact areas into intended and unintended.
- The article aims for completeness in the overview impact areas potentially affected by vehicle automation. The effort done in compiling the overview was substantial and the outcome was validated with couple of manuscripts not used in the process. Thus, the statement on completeness seems valid. However, there are a couple of items that may have been left outside the model on purpose but I was wondering if they would be needed for impact assessment later anyhow: First, you cannot have impacts without the use of technology. Thus, from the impact diagrams (Figures 3 & 4) I was wondering if “awareness” and “acceptance” should be visible, especially, if the model is aimed at decision makers. People need to be aware of technology and accept it before they start using it. This is an area where many stakeholders can influence the uptake of CCAM. Different levels of awareness and acceptance lead to evolving penetration rates and use of CCAM which affects the societal impacts. Another aspect, which is not included, is the impact on driving behavior (speed, headway, etc., “vehicle operations” in Innamaa *et al.* 2018b). The effects in these affect e.g. road capacity or emissions. Did you plan to include these in the algorithms of the next phase? Or how did you plan to take these topics into account? Some assumptions for them will be needed in the actual impact assessment phase. (I have a couple of additional suggestions to consider for Figure 4 below.)
- On page 7, in your description of the example in Figure 5, you say that “Figure 5 shows that AVs affect road safety…” and then you give examples that “they will have lower risk of being involved in an accident” but this in fact cannot be seen from Figure 5. Should you set the example with items visible in the graphics?
- On pages 8-9 you tell of “rebound effects”. Without knowing the direction of the impacts, it is hard to know whether they boost the impact or reduce it. Thus, I would reconsider calling it “rebound effect”, or at least I would add the word “potential” there to hint that these may potentially reduce the impact and therefore they are worth checking. It is hard to make a complete list of all potential rebounding effects. Potentially rebounding factors that are now not listed in Figure 4 and which are relevant include at least adaptation of driving style when AV user drives manually outside ODD (for at least users of SAE3 cars) and adaptation of the driving behavior of non-users in traffic with large penetration of AVs. If the driving outside ODD and of non-users are affected, they will have an impact, too.

This article presents the first step of very interesting work. It compiles the current knowledge on potential impact areas of CAV and presents them in structured way aiming to be generic for many CAV use cases. I look forward to seeing the model with quantified impacts.

Is the rationale for developing the new method (or application) clearly explained?

Yes

Is the description of the method technically sound?

Yes

Are the conclusions about the method and its performance adequately supported by the findings presented in the article?

Partly

If any results are presented, are all the source data underlying the results available to ensure full reproducibility?

Yes

Are sufficient details provided to allow replication of the method development and its use by others?

Yes

Reviewer Expertise:

Impact assessment, connected and automated driving, transport, mobility

I confirm that I have read this submission and believe that I have an appropriate level of expertise to confirm that it is of an acceptable scientific standard, however I have significant reservations, as outlined above.

# Response

**Published:** 2022-04-11

**Authors:** Diane Cleij

**Affiliations:** SWOV, The Netherlands

Dear Dr. Innamaa,

 Thank you for your comments, we feel they have improved the article significantly. In the text below we addressed your comments and indicated corresponding changes to the article text. Your comments are shown in bold font, our response is shown below in plain text and if any adjustments were made to the text this is shown in italic, with the exact adjustments underlined. We hope the revisions address your original reservations sufficiently.

 Kind regards,

 Diane Cleij  

**Reviewer Comment 1:**

**"The article is named “Impact assessment methodology for connected and automated vehicles”. However, in your own words “This paper presents the approach … to explore and define impacts and their interrelations as a starting point for quantitative impact assessment”. Specifically, the paper focuses on “impact areas” rather than on “impacts” as an assessment of the magnitude or even the direction of the impact is not included. Thus, the title does not really correspond to the content as the full impact assessment methodology is not presented but only the potential impact areas affected by CAVs. Therefore, I would suggest to rename the article as something like “Holistic overview of impact areas for CAV”.**

 We understand the confusion the title might cause and changed the title to:

*“ First steps towards a holistic impact assessment methodology for connected and automated vehicles”*  

**Reviewer Comment 2:**

**“In the abstract, you hint that “Results from the qualitative assessment … are presented”. However, if not even a direction of the impact is defined, can this be called “results of qualitative assessment”? I see this more as the identification of potential impact areas. Thus, I would recommend to clarify the content of the article for the abstract."**

 We understand the confusion the terminology might cause and changed the abstract phrase to:

 “ *The impact taxonomy and interrelations between impacts resulting from this project are presented and further steps needed to perform a quantitative evaluation of the impacts are discussed*”  

**Reviewer Comment 3:**

**“Terms “non-autonomous, partial and fully autonomous vehicles” are used on page 4. The term “automated” would be better."**

 Thank you for the suggestion, we corrected it in the text.  

**Reviewer Comment 4:**

**“On page 4 you state that “Beyond 25 years… mobility is expected to be “close to perfect”…”. Did you have a reference for this? Or is this a vision by LEVITATE? Such a strong statement would need clarification on this.”**

 We agree that this is a strong statement needing clarification. The statement indeed referred to an early vision within the LEVITATE project. However, this vision changed during the course of the project. As the statement also does not provide relevant information for this paper, we decided to remove it entirely.  

**Reviewer Comment 5:**

**“The article uses the taxonomy suggested by Chan, grouping the impact areas under vehicle users (direct), transport operations (systemic impacts) and society (wider) which seems well justified and works well in the outcome.”**

 We agree with the reviewer and do not think this comment calls for changes in the paper.  

**Reviewer Comment 6:**

**"On page 7 you discuss “intended and unintended impacts” stating that all direct, systemic and wider impacts are “intended” while behavioral adaptation represents “unintended” impacts. I am not sure if this can be said. For example, the car manufacturer intends to make the car safe, comfortable, etc. but most likely does not “intend” to affect road capacity. On the other hand, they may intend to build the car such that it increases the trust to technology or provides accessibility to wider demographic groups than traditional cars. Now these items are listed under “unintended impacts”. I agree that it is important to cover both intended and unintended impacts but without knowing whether an impact is positive or negative it is hard to group the areas into these categories. In fact, under one impact area there may be “intended” and “unintended” impacts, for safety there may be an “intended” reduction in many types of accidents but also some “unintended” new types of accidents caused by automation. Thus, I would be cautious in categorizing the impact areas into intended and unintended.”**

 Thank you for this remark. We agree that the use of the word “intended” does not correctly convey the meaning of primary and secondary impacts as they are presented in the paper. We adjusted the text as follows:

*“The impacts were classified as direct impacts, systematic impacts and wider impacts. These categories all refer to impacts that originate in automation technology, i.e. are stages of causal chains that start with technology. In addition, this technology could have secondary impacts. These impacts were modelled as behavioural adaptation and presented as a second impact diagram. The secondary impacts originate in changes in behaviour in response to the technology. The diagram showing primary impacts is shown in* *Figure 3* *, and one showing secondary impacts (behavioural adaptation; feedback) is presented in* *Figure 4* *. The impact generators in the second diagram are the direct impacts in the first diagram.”*  

**Reviewer Comment 7:**

**"The article aims for completeness in the overview impact areas potentially affected by vehicle automation. The effort done in compiling the overview was substantial and the outcome was validated with couple of manuscripts not used in the process. Thus, the statement on completeness seems valid. However, there are a couple of items that may have been left outside the model on purpose but I was wondering if they would be needed for impact assessment later anyhow: First, you cannot have impacts without the use of technology. Thus, from the impact diagrams (Figures 3 & 4) I was wondering if “awareness” and “acceptance” should be visible, especially, if the model is aimed at decision makers. People need to be aware of technology and accept it before they start using it. This is an area where many stakeholders can influence the uptake of CCAM. Different levels of awareness and acceptance lead to evolving penetration rates and use of CCAM which affects the societal impacts. Another aspect, which is not included, is the impact on driving behavior (speed, headway, etc., “vehicle operations” in Innamaa et al. 2018b). The effects in these affect e.g. road capacity or emissions. Did you plan to include these in the algorithms of the next phase? Or how did you plan to take these topics into account? Some assumptions for them will be needed in the actual impact assessment phase. (I have a couple of additional suggestions to consider for Figure 4 below.)”**

 Thank you for your comment.  Within the LEVITATE project constructs such as “awareness” and “acceptance” were taken into account when estimating future penetration rates of AVs. For each penetration rate the impacts as shown in the impact assessment schemes were estimated. These constructs are thus indirectly taken into account but not explicitly shown in the impact assessment schemes. These schemes do mention trust in technology, as we expect this to be strongly influenced by AV performance. Trust in technology can in turn influence acceptance and thus penetration rate. Within the project, however, we chose to use penetration rate as an input and evaluate the rest of the impact given this penetration rate. As for the driving behaviour impact, this was explicitly mentioned in the diagrams in earlier versions, but in the current version behaviour as a general category has been replaced by a set of more specific impact generators that refer to specific types of behaviour. Driving behaviour related to a single vehicle (with or without driver) is captured in the impact generator “level of automation”, while driving behaviour related to interaction between multiple vehicles is captured under “behaviour in interactions”.   

**Reviewer Comment 8:**

**“On page 7, in your description of the example in Figure 5, you say that “Figure 5 shows that AVs affect road safety…” and then you give examples that “they will have lower risk of being involved in an accident” but this in fact cannot be seen from Figure 5. Should you set the example with items visible in the graphics?”**

 This example refers in the first place to the arrow from impact generator “Level of automation” to wider impact  “Road safety”, here higher levels of automation could be hypothesized to reduce crash risk due to, for example, quicker reaction times. This effect likely increases with increasing level of automation as higher levels of automation could be assumed to, for example, have better situation awareness. This paragraph further mentions that vehicle connectivity influences road safety, which is indicated by the arrow from impact generator “Connectivity” to wider impact “Road Safety”. To make the relation between the example and the figure clearer we adjusted the text as follows:  

 “ *Figure 5* *shows that automated vehicles affect road safety directly (primary impact) via many routes, for example, they will probably have a lower risk of being involved in a crash than human driven vehicles , with the risk decreasing with increasing the level of automation. This impact is indicated in* *Figure 5* *with the arrow between “level of automation” and “road safety”.* *Especially if vehicles are able to communicate with each other, i.e. if they are connected (CAVs), the risk of a crash will probably be reduced. This additional improvement on road safety is indicated with the arrow between “connectivity” and “road safety”.* 

**Reviewer Comment 9:**

**"On pages 8-9 you tell of “rebound effects”. Without knowing the direction of the impacts, it is hard to know whether they boost the impact or reduce it. Thus, I would reconsider calling it “rebound effect”, or at least I would add the word “potential” there to hint that these may potentially reduce the impact and therefore they are worth checking. It is hard to make a complete list of all potential rebounding effects. Potentially rebounding factors that are now not listed in Figure 4 and which are relevant include at least adaptation of driving style when AV user drives manually outside ODD (for at least users of SAE3 cars) and adaptation of the driving behavior of non-users in traffic with large penetration of AVs. If the driving outside ODD and of non-users are affected, they will have an impact, too.”**

 We agree that the word “rebound” does not cover all feedback effects shown in Figure 6. We adjusted the text as follows.

*In addition, some potential feedback effects can be expected as shown in Figure 6. Such feedback effects can either amplify or reduce the original impact.*” The driving behaviour aspects you mention are currently captured under the impact generators “behaviour in interactions” and “level of automation”. We especially endorse the mentioned importance of including adaptive behaviour of non-AV-users to driving among AV, as this is an often overlooked effect. Within the LEVITATE project we have therefore conducted a driving simulation study investigating such effects and will soon publish a paper about the results.  

**Reviewer Comment 10:**

**“This article presents the first step of very interesting work. It compiles the current knowledge on potential impact areas of CAV and presents them in structured way aiming to be generic for many CAV use cases. I look forward to seeing the model with quantified impacts.”**

 Thank you, the quantified model is further developed in the European project LEVITATE. More information on the current status can be found on https://levitate-project.eu/
